# Sak4 of Phage HK620 Is a RecA Remote Homolog With Single-Strand Annealing Activity Stimulated by Its Cognate SSB Protein

**DOI:** 10.3389/fmicb.2018.00743

**Published:** 2018-04-24

**Authors:** Geoffrey Hutinet, Arthur Besle, Olivier Son, Stephen McGovern, Raphaël Guerois, Marie-Agnès Petit, Françoise Ochsenbein, François Lecointe

**Affiliations:** ^1^Micalis Institute, INRA, AgroParisTech, Université Paris-Saclay, Jouy-en-Josas, France; ^2^Institute for Integrative Biology of the Cell (I2BC), IBITECS, CEA, Centre National de la Recherche Scientifique, Université Paris-Sud, Université Paris-Saclay, Gif-sur-Yvette, France

**Keywords:** Sak4, Rad51 paralog, RecA, strand exchange protein, annealase, SSB, recombineering, bacteriophage

## Abstract

Bacteriophages are remarkable for the wide diversity of proteins they encode to perform DNA replication and homologous recombination. Looking back at these ancestral forms of life may help understanding how similar proteins work in more sophisticated organisms. For instance, the Sak4 family is composed of proteins similar to the archaeal RadB protein, a Rad51 paralog. We have previously shown that Sak4 allowed single-strand annealing *in vivo*, but only weakly compared to the phage λ Redβ protein, highlighting putatively that Sak4 requires partners to be efficient. Here, we report that the purified Sak4 of phage HK620 infecting *Escherichia coli* is a poorly efficient annealase on its own. A distant homolog of SSB, which gene is usually next to the *sak4* gene in various species of phages, highly stimulates its recombineering activity *in vivo. In vitro*, Sak4 binds single-stranded DNA and performs single-strand annealing in an ATP-dependent way. Remarkably, the single-strand annealing activity of Sak4 is stimulated by its cognate SSB. The last six C-terminal amino acids of this SSB are essential for the binding of Sak4 to SSB-covered single-stranded DNA, as well as for the stimulation of its annealase activity. Finally, expression of *sak4* and *ssb* from HK620 can promote low-level of recombination *in vivo*, though Sak4 and its SSB are unable to promote strand exchange *in vitro*. Regarding its homology with RecA, Sak4 could represent a link between two previously distinct types of recombinases, i.e., annealases that help strand exchange proteins and strand exchange proteins themselves.

## Introduction

Homologous recombination (HR) is a key DNA repair mechanism in all living organisms and a major force for evolution. Placed at the center of this process, recombinases play key roles in the search for homology. In Bacteria, this recombinase is RecA, and its mechanism of action is well known, particularly in *Escherichia coli* (reviewed in Bell and Kowalczykowski, 2016). Rad51 and RadA are RecA orthologs in Eukarya and Archaea, respectively, sharing homology, ubiquity, and mechanism of action with RecA (Seitz et al., 1998; Krogh and Symington, 2004; Haldenby et al., 2009; Krejci et al., 2012). Briefly, all these recombinases bind single-stranded DNA (ssDNA), form a pre-synaptic filament on it, invade a double-stranded DNA (dsDNA) displaying sequence complementarity and provoke the reciprocal exchange of strands.

Phylogenetically, the family of RecA-like proteins can be divided into three branches, two of them being composed of the well conserved RecA and Rad51/RadA orthologs, while the third branch is made up of divergent RecA or Rad51 paralogs, with distinct but less well characterized roles compared to RecA and Rad51 (Lin et al., 2006). Some Bacteria contain a *recA* paralog, named *sms* or *radA* (Cooper et al., 2015; Cooper and Lovett, 2016), all designated as *sms* below, to avoid confusion with the archaeal *radA*. In Archaea, two families of paralogs have been described and characterized biochemically: RadB and RadC (Komori et al., 2000; McRobbie et al., 2009). Several Rad51 paralogs were identified in Eukarya. The most studied are Rad55 and Rad57 in fungi, as well as Csm2 and Psy3 proteins of the Shu-complex, or RAD51B, RAD51C, RAD51D, XRCC2, and XRCC3 proteins in mammals (for reviews see Suwaki et al., 2011; Karpenshif and Bernstein, 2012).

Rad51/RecA paralogs share with RecA only the core domain, responsible for two key functions: ATP hydrolysis and ssDNA binding. Around this core domain, paralogs sometimes have N- or C-terminal extensions, but they all miss a ~70-residues domain present in the C-terminus of RecA and in N-termini of Rad51/RadA, necessary for the secondary DNA-binding site of the recombinase, which appears critical in the strand exchange reaction (Lusetti et al., 2003; De Vlaminck et al., 2012). These paralogs participate in various ways in recombination events (Komori et al., 2000; Takata et al., 2001; Godin et al., 2013), but none of them seems able to form nucleofilaments by themselves, nor promote the strand invasion reaction typical of *bona fide* RecA proteins.

Among phages (viruses infecting bacteria) with large dsDNA genomes (>20 kb), a majority contains their own genes coding for HR proteins. In a systematic study performed in 2010, among a collection of 325 genomes (>20 kb), 191 (60%) contained homologous recombination genes (Lopes et al., 2010). Seven percent of these 191 genomes (mostly virulent phages with >100 kb genome) contained a gene coding for a protein with sequence and structure similarities to RecA, the best characterized being UvsX of phage T4 infecting *E. coli* (Liu and Morrical, 2010 for review). Moreover, UvsX forms nucleofilaments structurally similar to those formed by RecA (Stasiak and Egelman, 1994). UvsX is important for the late replication stage of the T4 phage, during which replication initiates by recombination (for review see Kreuzer and Brister, 2010).

Remarkably, HR in phages relies more often on a Rad52-like single-strand annealing (SSA) protein (Ploquin et al., 2008; Erler et al., 2009; Lopes et al., 2010) than on RecA-like, as among 191 genomes having HR functions, 50% contained a gene coding for a Rad52-like SSA protein (SSAP, Lopes et al., 2010). One of them is particularly well characterized: Redβ of bacteriophage λ recombines DNA without any help from RecA, and with relaxed fidelity (Martinsohn et al., 2008; De Paepe et al., 2014). In addition, it performs *in vivo* the so-called recombineering reaction whereby a ssDNA molecule is annealed into the bacterial chromosome by complementarity, most likely behind the replication fork and with a preference for the lagging strand template (Murphy, 2016 for review). Of interest, RecA is unable to perform such a reaction.

Besides this set of phages equipped with well characterized Rad52- or RecA-like enzymes, many code for a “core-only RecA” (23% of the 191 analyzed), resembling RAD51 paralogs and belonging to the Sak4 family, on which much less is known. It should not be confused with Sak and Sak3, both Rad52-like SSAP (see below). Genes coding for Sak4 are present on medium-sized genomes of both virulent (N4-like) and temperate phages (such as HK620), infecting both Gram- and Gram+ bacteria, with a large representation among *Staphylococci* and *Streptococci* phages (Lopes et al., 2010; Delattre et al., 2016). The *sak4* gene was first identified in a phage/bacterium co-evolution experiment, whereby mutants of phage 31 were isolated that were able to infect a *Lactococcus lactis* phage resistant strain harboring the abortive infection system *abiK* (Bouchard and Moineau, 2004). The phage mutations were all in a gene of previously uncharacterized function, that was named *sak4* (sak stands for suppressor of AbiK). As AbiK was known to target phage SSAP genes such as *sak* and *sak3* of the Rad52-like family, it was suggested that Sak4 was a new kind of HR protein, with no homology to Rad52. Later on, we reported that expression of the *sak4* gene located in the genome of phage PA73 of *Pseudomas aeruginosa* led to slight increase of recombination events in a recombineering assay in *E. coli*, suggesting it was a SSAP (Lopes et al., 2010).

For Rad51, RecA and UvsX, the main obstacle to overcome in order to reach single-strand DNA and initiate homology search is the single strand binding protein (RPA for Rad51, SSB for RecA, Gp32 for UvsX on phage T4), a phenomenon referred to as the SSB barrier effect (Beernink and Morrical, 1999). To help displacing SSB, various recombination mediator proteins are used, such as Rad52 in Eucarya, RecFOR, DprA, or RecBCD in Bacteria, or UvsY in phage T4 (Beernink and Morrical, 1999; Mortier-Barrière et al., 2007). However, the role of SSB in RecA-promoted strand exchange *in vitro* is dual, as once RecA is nucleated onto DNA, SSB_*Ecoli*_ facilitates its polymerization by suppressing DNA secondary structures (Kowalczykowski and Krupp, 1987). In addition, it is proposed that SSB_*Ecoli*_ catches the displaced strand during the D-loop formation, to stabilize the recombination intermediate (Cox, 2007; Shereda et al., 2008). With respect to phage SSAP proteins, no particular involvement, positive or negative, of SSB has been reported. Many phages contain their own *ssb*-like gene, with sometimes a large divergence to bacterial *ssb* (Szczepanska et al., 2007).

Besides their role in recombination, phage SSAP have been regularly suggested to play also a role in phage replication, but results were contradictory, and phenotypes moderate. Recently however, Sak and Sak4 proteins, synthetized by *Staphylococcus* phages 80α and Φ11 respectively, were shown to be essential for phage growth, and phage genome multimers were barely produced in their absence. Interestingly, phage *ssb* mutants had similar, although less pronounced phenotypes, suggesting a genetic interaction between Sak/Sak4 and their cognate phage SSB (Neamah et al., 2017).

Here, we report on the genetic and biochemical characterization of the Sak4 protein (formerly HkaL) produced by phage HK620 infecting *E. coli*. We show that this core-only RecA has SSA activity *in vitro*, and recombineering capacity *in vivo*, and characterize its need for its cognate SSB for full activity.

## Results

### The purified Sak4 protein of phage HK620 binds ssDNA in an ATP-dependent way

**Figure 1:**
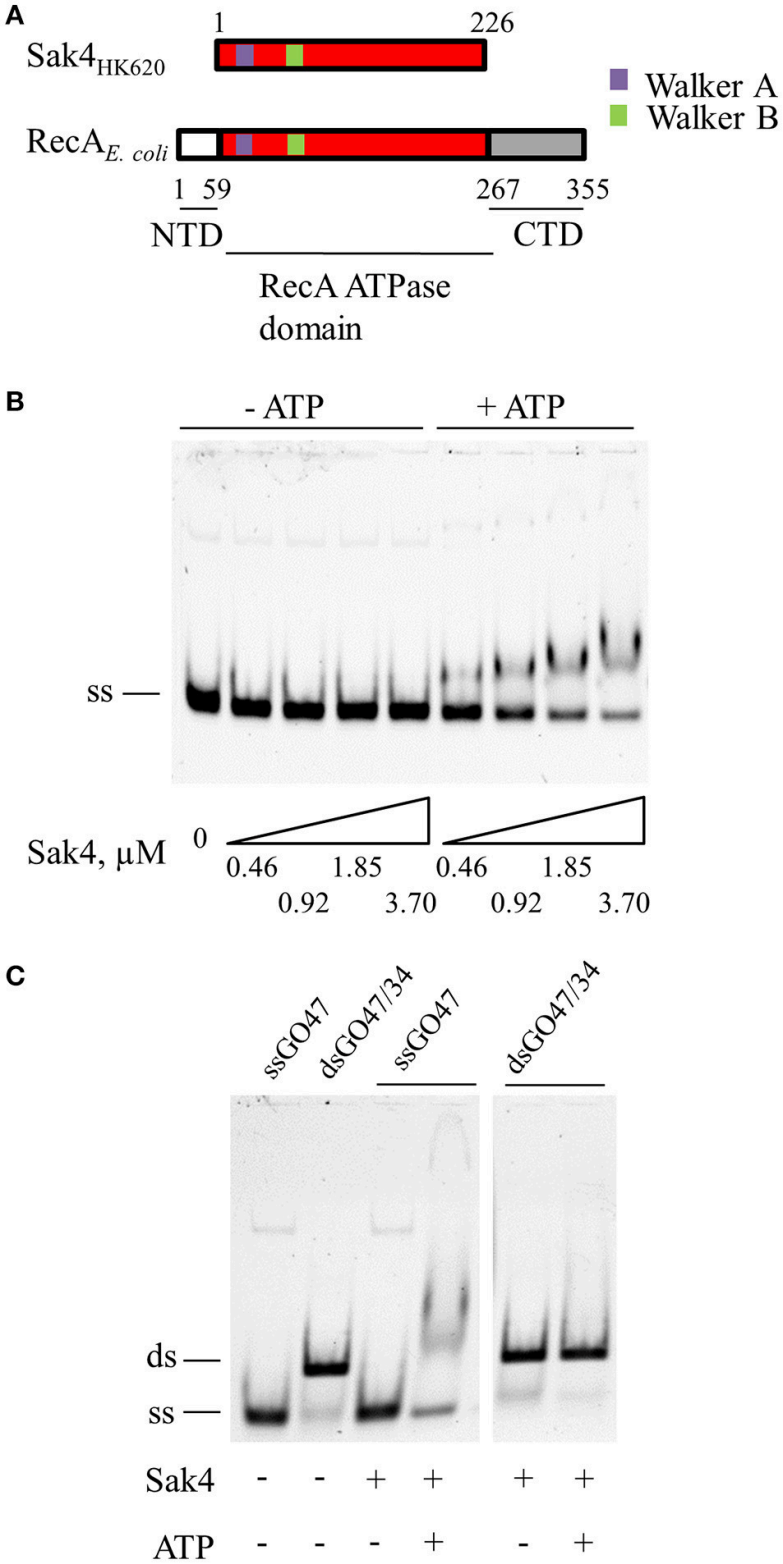
Sak4 is an ATP-dependent ssDNA binding protein. **(A)** Domain positions in Sak4 from phage HK620 and *E. coli* RecA. Sak4 displays homology with the central domain of RecA (in red, labeled “RecA ATPase domain”) and is devoid of the secondary C-terminal DNA-binding domain of RecA (in gray, labeled “CTD”). **(B)** ssDNA binding assay. Ten nanomolars of oligonucleotide GO47 (ss) was mixed with the indicated Sak4 concentration in the presence or not of 1 mM ATP for 15 min at 30°C, and then loaded on a 5% acrylamide/bis-acrylamide (19:1) gel. **(C)** dsDNA binding assay. Ten nanomolars of GO47 (band labeled “ss”), or 10 nM of dsDNA formed by the annealing of GO47 and GO34 prior to the EMSA (band labeled “ds”), were incubated (+) or not (−) with 3.7 μM Sak4 and with (+) or without (−) 1 mM ATP and analyzed as in **B**.

Our first *in vivo* characterization of a Sak4 (from phage PA73, infecting *P. aeruginosa*), suggested a behavior more related to Rad52-like proteins than to RecA, as it permitted recombineering, although at a very low level compared to Redβ (Lopes et al., 2010). This prompted us to investigate the biochemical properties of Sak4, side by side with Redβ, RecA, and UvsX. The purified Sak4 protein of phage PA73 proved insoluble (not shown), so we focused on a Sak4 (NP_112045) from an *E. coli* infecting phage: HK620 (Lopes et al., 2010). This protein displays homologies with the *E. coli* RecA protein central domain (Figure 1A). The purified Sak4 from HK620 was devoid of any tag, since different tagged versions of this protein formed inclusion bodies in *E. coli*. Redβ and UvsX were expressed as GST fusions, and used after protease removal of the tag (see section Materials and Methods), while a commercial preparation of *E. coli* RecA was used. Coomassie stained gels of all proteins used below are shown in Supplementary Figure S1.

The affinity of Sak4 for ssDNA was tested using a gel shift assay. Increasing concentrations of Sak4 were incubated in the presence of 10 nM of 5′-Cy5 labeled, 81-mer oligonucleotide (GO47), and products were separated on gel. Sak4 shifted the ssDNA only if ATP was added in the reaction buffer (Figure 1B). ADP did not allow Sak4 binding to ssDNA (not shown). A 1.85 μM concentration of Sak4 was needed to shift about half of the oligonucleotide in the presence of ATP, indicating that affinity of Sak4 for ssDNA is quite low. In contrast, dsDNA (10 nM) was not shifted by 3.7 μM Sak4, neither with nor without ATP (Figure 1C). We conclude that Sak4 is an ATP-dependent ssDNA binding protein, a property reminiscent of UvsX (Maher and Morrical, 2013).

### Sak4 is an ATP dependent annealase

**Figure 2:**
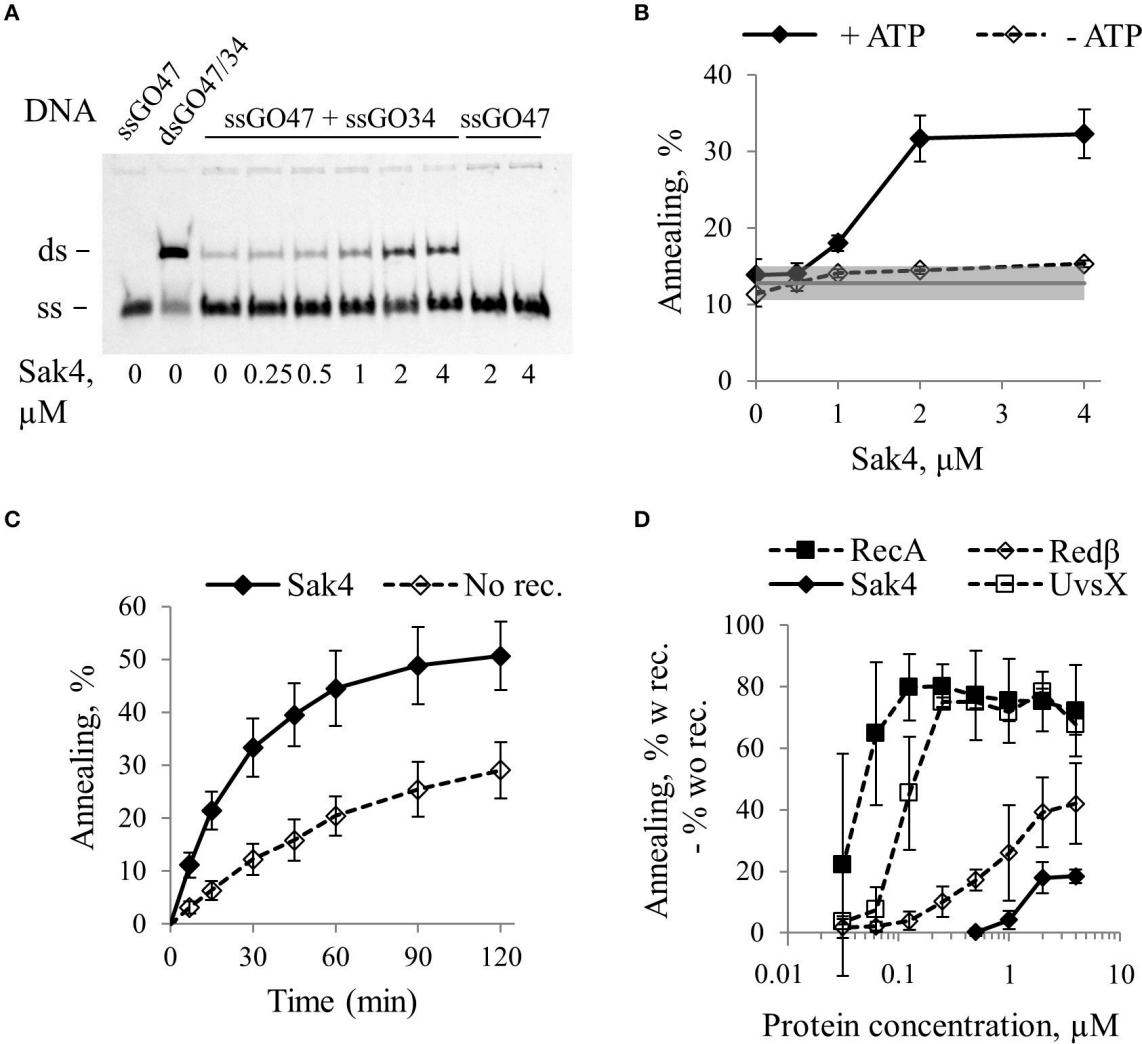
Sak4 has single strand annealing activity *in vitro*. **(A)** Representative gel of an SSA assay. Ten nanomolars of oligonucleotide GO47 was pre-incubated for 15 min with variable concentrations of Sak4, as indicated below the gel in a buffer containing 1 mM ATP. Reactions were started by addition (ssGO47 + ssGO34) or not (ssGO47) of 10 nM of the complementary oligonucleotide GO34 and incubated for 30 min at 30°C. Products of the reaction were separated by PAGE. First lane: GO47 oligonucleotide alone (band labeled “ss”). Second lane: dsDNA formed by the annealing of GO47 and GO34 (upper band, labeled “ds”). **(B)** Quantification of the SSA activity of Sak4 on the GO47/GO34 pair of oligonucleotides. Reactions were performed as described in **A** (ssGO47 + ssGO34) except that reaction buffer contains or not 1 mM ATP, as indicated. DNA bands were quantified to determine the annealing efficiency as a function of Sak4 concentration. The mean values and standard deviations from three experiments are given. The gray line and gray band correspond respectively to the mean value and standard deviation of the spontaneous annealing (without Sak4) in these experiments. **(C)** Kinetics of annealing. Reactions were performed in a buffer containing the two complementary ssDNA and 1 mM ATP with (Sak4) or without (No Rec.) 1.85 μM Sak4. The mean values and standard deviations from three experiments are given. **(D)** Comparison of annealing efficiencies of Sak4, RecA, Redβ, and UvsX. Reactions were performed for 7 min (RecA, Redβ, and UvsX) or 30 min (Sak4) at 30°C in different buffers (see text). Each point corresponds to the % of annealing of the two complementary ssDNA in the presence of the recombinase subtracted from the % of spontaneous annealing. Average and standard deviations are given for at least 2 repeats.

The SSA activity of Sak4 was monitored by incubating the protein with two complementary 81-mer oligonucleotides (5′-Cy5-labeled GO47, and unlabeled GO34) in the presence of ATP. After protein removal, DNA products were analyzed by native polyacrylamide gel electrophoresis (Figure 2A). In the absence of Sak4 (third lane from the left), two DNA bands were observed after 30 min of incubation at 30°C. The lower band (ss) corresponded to the GO47 oligonucleotide and the upper band (ds) corresponded to the dsDNA molecules formed by spontaneous annealing of the two oligonucleotides. The amount of GO47 spontaneously annealed with GO34 in this assay was typically around 10–15%. In the absence of GO34, and due to the deproteinization of the samples before separation of DNA molecules on gel, Sak4 did not change the migration pattern of GO47 (see the last two lanes), therefore excluding the presence of confounding retarded nucleoprotein species in this experiment. In the presence of GO47 and GO34, addition of Sak4 led to increased formation of the dsDNA molecules, with a maximal annealing around 30–35% with 2 μM of Sak4. Annealing efficiency was quantified in the presence or absence of ATP (Figure 2B). As expected from the DNA binding experiment (Figure 1), SSA mediated by Sak4 required ATP.

Time course experiments in the presence of 1.85 μM Sak4 and ATP showed that Sak4-promoted annealing was about 3 times more efficient than spontaneous annealing during the first 30–45 min of the reaction (Figure 2C). The reaction was incomplete (never more than 25% above spontaneous reaction), and this was not due to ATP exhaustion, since adding an ATP regeneration system did not improve the annealing efficiency (not shown).

We compared the SSA activity of Sak4 with those of RecA from *E. coli*, Redβ from λ and UvsX from T4. To optimize SSA for Redβ, UvsX, and RecA, NaCl concentration was lowered from 150 to 10 mM, and ATP was removed for RecA and Redβ. Incubation time was 30 min for Sak4, and 7 min for the other proteins (reactions almost complete). Quantification of the SSA activities of these proteins (after removal of spontaneous annealing) are reported Figure 2D. In our conditions, 125 nM of RecA was sufficient to obtain a maximal annealing of ~80%. For Redβ, 2 μM of the protein was the minimal concentration leading to a maximal annealing of ~40%. UvsX activity behaved like RecA, except that 0.5 μM was required for maximal SSA (Figure 2D). Finally, as described above, 2 μM of Sak4 was only able to increase the annealing of the complementary oligonucleotides to 20% above the spontaneous annealing. Incubation time of 30 min of complementary ssDNA with the GST protein (that does not bind to DNA) did not lead to higher amount of product compared to the spontaneous annealing (Supplementary Figure S2). This demonstrates that the SSA mediated by Sak4 (and the other recombinases) is specific for this protein and not due to a protein concentration effect. Sak4, RecA and Redβ were also able to anneal up to 20% diverged oligonucleotides (Supplementary Figure S4).

We conclude that Sak4 is an annealase displaying a weak SSA activity on its own compared to RecA, UvsX, and Redβ, that parallels its weak efficiency to promote recombineering *in vivo* (Sak4 from the phage PA73 Lopes et al., 2010, and see below).

### Genome of phages with *sak4* often contain a distant *ssb* homolog nearby

**Figure 3:**
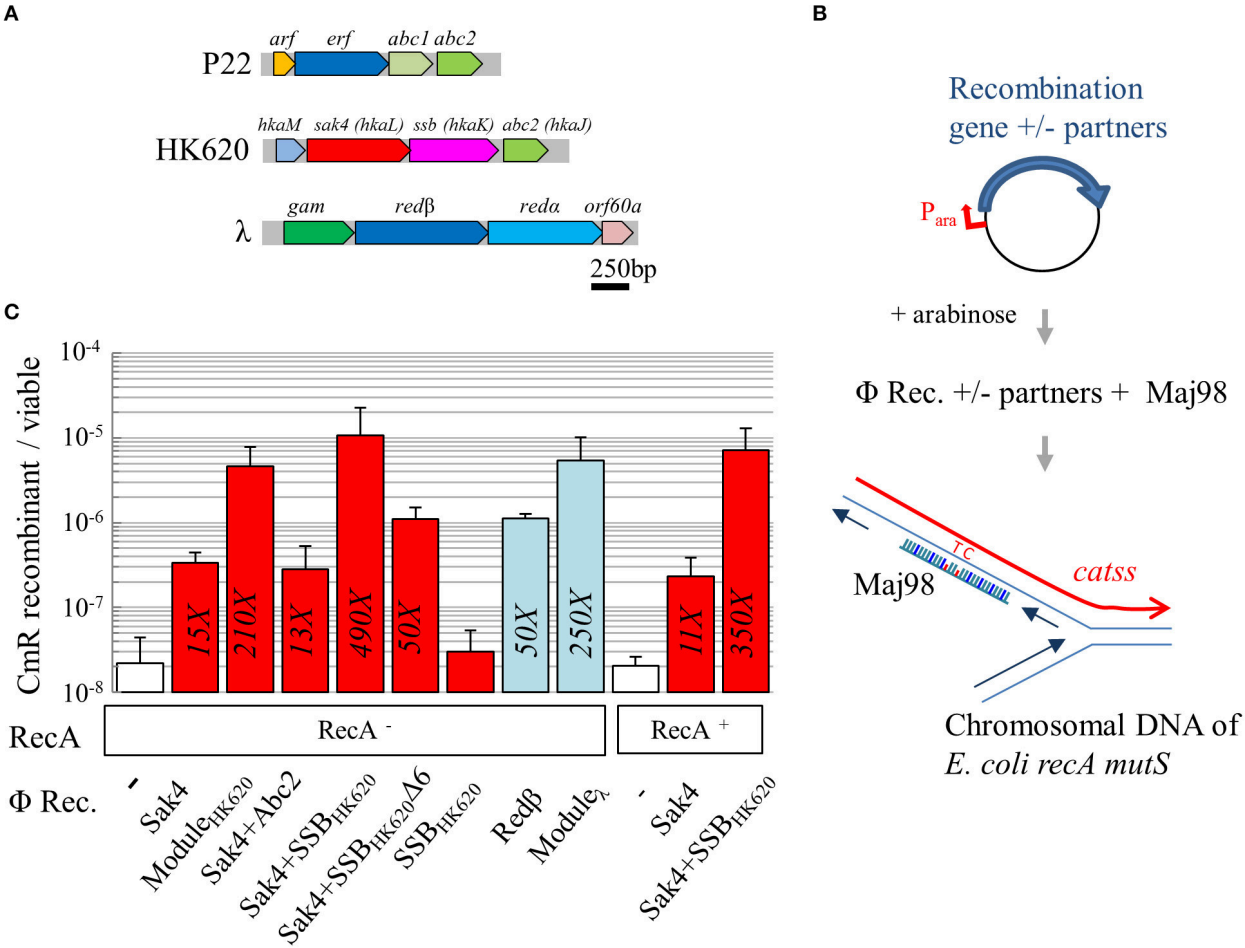
Sak4 has recombineering activity stimulated by SSB_HK620_. **(A)** Genetic organization of the recombination module in three phages. In phage P22, *arf is* of unknown function, *erf* codes for a Rad52-like recombinase, and *abc1* and *abc2* code for RecBCD hijackers. In phage HK620, *hkaM* is of unknown function, *hkaL* or *sak4*, codes for a core-only RecA recombinase, *hkaK* or *ssb*, codes for a single strand binding protein, and *abc2* is homologous to P22 *abc2*. In phage λ, *gam* codes for an inhibitor of RecBCD, *red*β codes for a Rad52-like SSAP distantly related to P22 *erf*, *red*α codes for the λ exonuclease, and *orf60a* is of unknown function. **(B)** Principle of the recombineering assay: the *E. coli recA mutS* strain, carrying a plasmid coding for a recombinase and/or other proteins, under the control of an arabinose inducible promoter, is electroporated with Maj98, an 81-mer oligonucleotide, complementary to a mutated *cat* gene (*catss*) located on the chromosome, except for two positions restoring the wild type *cat* gene. Integration of the oligonucleotide at the chromosomal replication fork confers chloramphenicol resistance to the bacterium. **(C)** Recombineering efficiency of various phage recombinases (“Φ Rec.” legend) in a RecA^−^ or RecA^+^ background (“RecA” legend). The first, white bar represents spontaneous recombineering obtained in the RecA^−^ strain with the empty vector (MAC1879). Recombineering efficiency of *sak4* alone (strain MAC2089), together with its 3 neighboring genes (“Module_HK620_,” see panel **A**, strain MAC1798), *sak4* and *abc2* (MAC1897), *sak4* and *ssb*_HK620_ (MAC1895), *sak4* and *ssb*_HK620_Δ6 (MAC2134), and *ssb*_HK620_ alone (MAC1922) are shown as red bars. *red*β alone (MAC1894) or together with its three neighboring genes (“Module_λ_,” MAC1801) are shown as blue bars. Finally, experiments performed in RecA^+^ isogenic strains (MAC1774 and derivatives) are shown. The fold increases, relative to the empty vector control, is indicated in each bar only if the recombination efficiency is statistically different from the background level (as determined by a student *t*-test, *p* < 5%). Experiments were performed at least three times, and error bars indicate the standard deviation around the mean.

The weak activity of Sak4 suggested that this protein could require one or several other protein partners to be fully efficient. In the original annotation of the phage HK620 genome (Clark et al., 2001), four predicted genes were suspected to participate in homologous recombination by comparison with the *Salmonella enterica* subsp. *typhimurium* phage P22 genome organization (Figure 3A). These genes were initially annotated as *arf* (*hkaM), erf* (*hkaL), abc1* (*hkaK*), and *abc*2 (*hkaJ*) respectively, similarly to P22. As described previously, *hkaL* codes for a Sak4 (Lopes et al., 2010), which displays sequence homology with the central domain of RecA (Figure 1A) and is not homologous to Erf of P22. For the three surrounding genes, upon closer inspection, only Abc2 (NP_112043) shares homology with the Abc2 protein encoded by P22 (94% identical amino acids). This protein functions as a hijacker of RecBCD, converting this dsDNA exonuclease into a 5′-3′ exonuclease that prepares DNA extremities for recombination (Murphy, 2000). The Arf protein (NP_112046) is orphan, and has no homology to Arf of P22, so we name it here HkaM. Finally, the Abc1 protein (NP_112044) has no similarity to Abc1 of P22, despite occupying a similar genetic position in the recombination module. We found however that this protein (corresponding to the Pfam DUF669 domain) was a distant homolog of the SSB proteins of phages N4, Lc-Nu and PhiAT3 (see Materials and Methods and Supplementary Figure S3). All these phage *ssb* are next to a *sak4* gene. The reverse is not true, but up to 80% of the *sak4* genes are found next to an *ssb* gene (Supplementary Table S5). *In vitro* analyses will show below that HkaK/Abc1_HK620_ is indeed an SSB, and for simplicity we name it already SSB_HK620_.

### SSB_HK620_ stimulates Sak4 recombineering *in vivo* and this stimulation is dependent on its last six C-terminal residues

A sensitive *in vivo* recombineering assay, minimizing the background level of spontaneous revertants, was designed. *E. coli* strain MAC1802 was transformed by Maj98, an 81-mer oligonucleotide correcting the two consecutive stop codon mutations of its *cat* gene (*catss* allele) on the chromosome. Maj98 was complementary to the lagging strand template, relative to the chromosomal replication fork (Figure 3B). Recombineering efficiency was estimated by the ratio of chloramphenicol resistant (CmR) to total bacterial count. Strain MAC1802 was also mutated for *recA*, to exclude any contribution of RecA on the effects observed, and for *mutS*, to prevent inhibition of recombineering due to mismatch detection. Plasmids containing various genes cloned under an arabinose inducible promoter were introduced into this strain. Gene expression was induced by arabinose addition 45 min prior harvest and cell washes for electroporation. In strain MAC1879 (MAC1802 containing the pJA4 plasmid used as a control), the spontaneous occurrence of CmR revertants was below the detection level (10^−9^). A mock experiment of transformation with Maj98 oligonucleotide in MAC1879 showed that spontaneous recombineering occurs *in vivo*, at a frequency of 2.2 × 10^−8^ (Figure 3C, first value, white bar; all values are reported in Supplementary Table S6). Redβ from the phage λ was used as a positive control. A 50-fold increase relative to the spontaneous annealing was observed upon *red*β expression (Figure 3C, blue bars), and 250-fold with pKD46 (5.4 × 10^−6^), the original plasmid used for recombineering (Datsenko and Wanner, 2000) containing the whole module of recombination genes of phage λ (see the module map Figure 3A).

Recombineering was increased 15-fold with *sak4* expressed from the pGH3 plasmid, compared to the empty vector control (Figure 3C). To test whether the recombineering activity of Sak4 could be stimulated by an accessory protein, the pJA17 plasmid containing the whole predicted recombination module (“Module_HK620_”, containing the *sak4, ssb*_HK620_, *abc2*, and *hkaM* genes, Figure 3A) was tested. A frequency of recombinants 210-fold over the spontaneous annealing was obtained with this construct. We next investigated whether a single of these genes was sufficient to induce this stimulating effect. Co-expression of *abc2* and *sak4* (from pGH20) had no effect, while arabinose induction of the *sak4*-*ssb*_HK620_ in the pGH19 plasmid restored completely the phenotype observed with the all three surrounding genes (Figure 3C, compare the second and fourth red bars). Importantly, the pGH21 plasmid producing the SSB_HK620_ protein alone did not increase the spontaneous annealing level (Figure 3C).

The acidic C-terminal tail of many bacterial SSB is critical for protein-protein interactions with RecO, PriA and several other proteins involved in genome maintenance (Shereda et al., 2008; Costes et al., 2010). The last 13 amino acids of the C-terminal tail of SSB_HK620_ encompass three aspartate residues, and the C-terminal sequence NDYPPF is similar to that of SSB_*Ecoli*_ (DDDIPF). We asked therefore whether this NDYPPF motif could be involved in the stimulation of the recombineering activity of Sak4. The pOS10 plasmid coding for Sak4 and a mutated SSB_HK620_ deleted for its six C-terminal residues (named hereafter SSB_HK620_Δ6) led to a 10–fold reduction of recombineering, compared to the *sak4-ssb*_HK620_ construct. This indicates that the NDYPPF C-terminal tail of SSB_HK620_ is strongly involved for recombineering stimulation. The recombineering was still 3-fold higher in the strain co-expressing *sak4* and *ssb*_HK620_Δ6 than *sak4* alone, suggesting that a minor part of the stimulating effect is independent on the C-terminal tail of SSB_HK620_.

In an isogenic, RecA^+^ background, the recombineering efficiency of Sak4 was not further enhanced at this *catss* locus, suggesting that RecA does not further facilitate the process (Figure 3C). Sak4-mediated recombineering was tolerant to 12% divergence at the *catss* locus, like Redβ (Supplementary Figure S4).

We noticed that recombineering efficiency was low at the *cat*_*SS*_ locus compared to published values at the *galK* locus in other strains (Costantino and Court, 2003). Indeed, at the *galK* locus (strain G205, a *recA* derivative of HME57, Supplementary Table S1) a recombineering frequency of 2.2 × 10^−3^ was reached with pKD46, using an oligonucleotide of a similar length and targeted to the lagging strand, a value which was 4,000-fold higher than the spontaneous annealing in this strain (see Supplementary Table S6). In contrast, in this more optimal context for the λ recombination module, expression of *sak4* from the pGH3 plasmid led to a slight 3-fold increase of the recombineering compared to the spontaneous recombineering (Supplementary Table S6). A stimulating effect of the expression of the *ssb*_HK620_ with *sak4* was observed again (36-fold increased compared to the spontaneous recombineering).

The recombineering activity of Sak4 was also tested in the ER2566 strain allowing expression of genes cloned on a plasmid under the strong T7 promoter. To this end, we used an oligonucleotide targeting the *rpoB* locus and conferring, after recombineering, resistance to the rifampicin. The same global effect was observed: rifampicin resistant (RifR) recombinants yield was 4-fold and 20-fold increased compared to the spontaneous annealing for Sak4 alone and the Sak4-SSB_HK620_ pair, respectively (Supplementary Table S6).

We conclude that Sak4 displays a low recombineering activity *in vivo* that is stimulated by the presence of its neighboring gene coding for SSB_HK620_, regardless the targeted locus and with similar efficiencies in different genetic background. This stimulating effect mainly involves the NDYPPF C-terminal residues of SSB_HK620_.

### SSB_HK620_ is a *bona fide* SSB that stimulates Sak4 annealing, depending on the presence of its C-terminal tail

**Figure 4:**
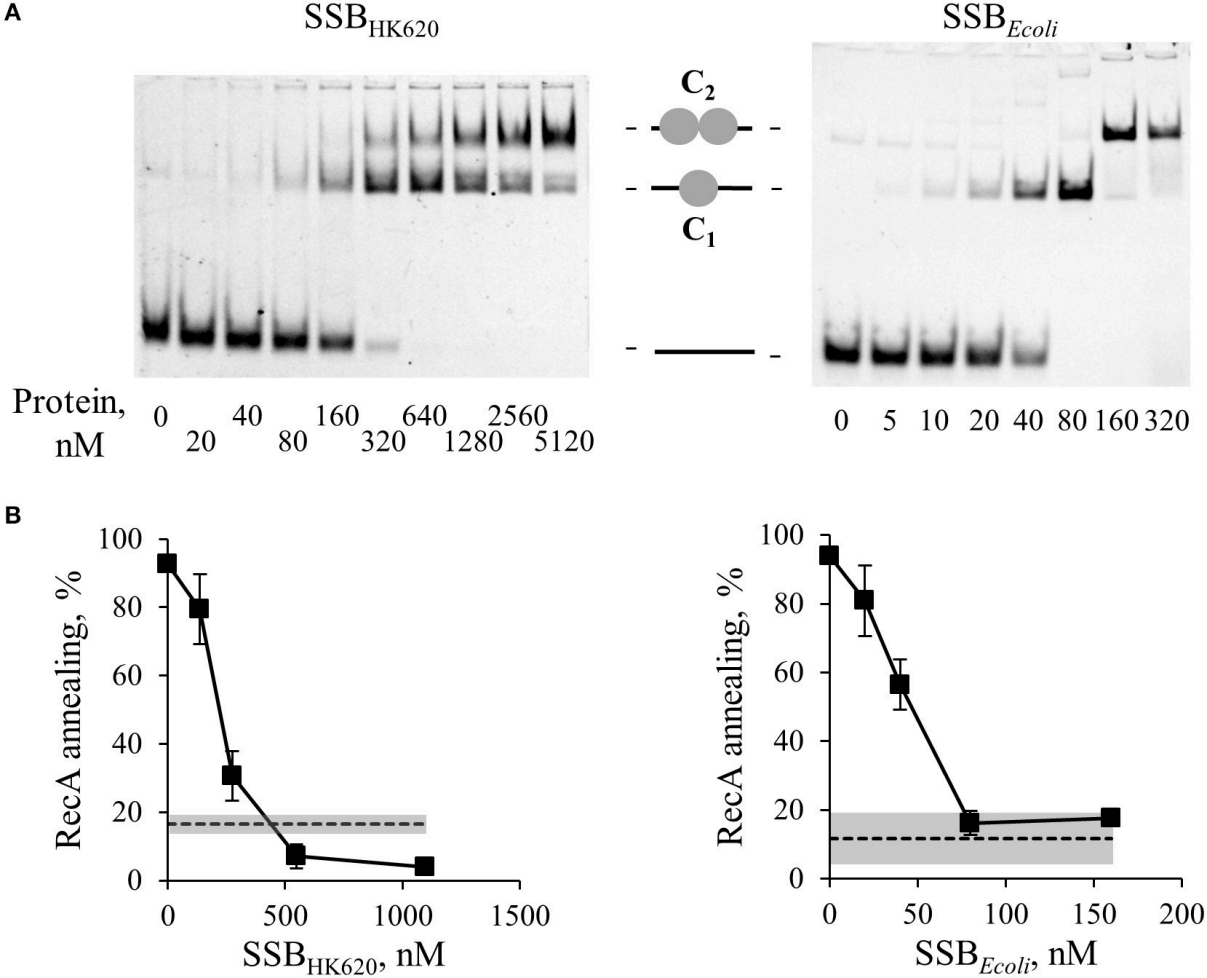
SSB_HK620_ shares similar DNA binding properties with *E. coli* SSB and inhibits the SSA mediated by RecA. **(A)** Gel shift of ssDNA by SSB_HK620_ or *E. coli* SSB, as indicated above the gels. GO47 (10 nM) was mixed with SSB proteins at the indicated concentrations for 15 min at 30°C, and then loaded on a 5% acrylamide/bis-acrylamide (19:1) gel. Sequential binding of one (1 gray circle, complex C1) then two SSB homotetramers (2 gray circles, complex C2) on the ssDNA molecule (black bar) is illustrated. **(B)** Effect of these SSB proteins on the ability of RecA to promote SSA. Increased concentrations of SSB proteins (abscissa), were pre-incubated 15 min at 30°C with GO47 (10 nM) and an additional 15 min with 1.85 μM of RecA. The SSA reaction was then started by the addition of the complementary GO34 (10 nM). Experiments were done at least three times; mean values and standard deviations are given. Dashed lines and gray bands correspond respectively to the mean values and standard deviations of the spontaneous annealing (without proteins) in these experiments.

SSB-bound ssDNA prevents RecA-mediated strand exchange, and constitutes a barrier to SSA (Beernink and Morrical, 1999). It seemed therefore counter intuitive that a phage SSB would stimulate Sak4 recombineering *in vivo*. We first purified SSB_HK620_ (without tag)_._ The protein (molecular weight (MW) of the monomer = 21.3 kDa) was eluted from a size exclusion chromatography (SEC) column with an apparent MW~95 kDa (Supplementary Figure S5), suggesting that SSB_HK620_ is a tetramer, like SSB_*Ecoli*_. We next tested whether this protein behaved as an SSB *in vitro*. Its affinity for ssDNA was compared to that of SSB_*Ecoli*_ in a gel shift assay. As expected, SSB_*Ecoli*_ formed two complexes with the GO47 oligonucleotide, corresponding to the sequential binding of a first (complex C1), then a second homotetrameric SSB_*Ecoli*_ (complex C2) to the ssDNA substrate (Figure 4A). With SSB_HK620_, two shifted species were also observed, which we interpreted as the formation of complexes C1 and C2, by analogy with SSB_*Ecoli*_ (Figure 4A). The affinity of SSB_*Ecoli*_ for ssDNA was higher than that of SSB_HK620_: to shift half of the ssDNA, 40 nM SSB_*Ecoli*_ was sufficient, whereas 4-fold more SSB_HK620_ was needed (between 160 and 320 nM). Finally, we tested if SSB_HK620_ was able to act as a barrier to the RecA-mediated SSA. Increasing concentrations of SSB_HK620_ were incubated with the oligonucleotide GO47 (10 nM final concentration) for 15 min at 30°C prior the addition of RecA (1.85 μM final concentration) for an additional incubation of 15 min at 30°C. The complementary GO34 oligonucleotide was then added and reactions were analyzed after 30 min. Experiments using SSB_*Ecoli*_ instead of SSB_HK620_ were done in parallel for comparison. Under these conditions, SSB_HK620_ and SSB_*Ecoli*_ inhibited the SSA activity of RecA (Figure 4B). We conclude that SSB_HK620_ shares common characteristics with SSB_*Ecoli*_, despite a lower affinity for ssDNA.

**Figure 5:**
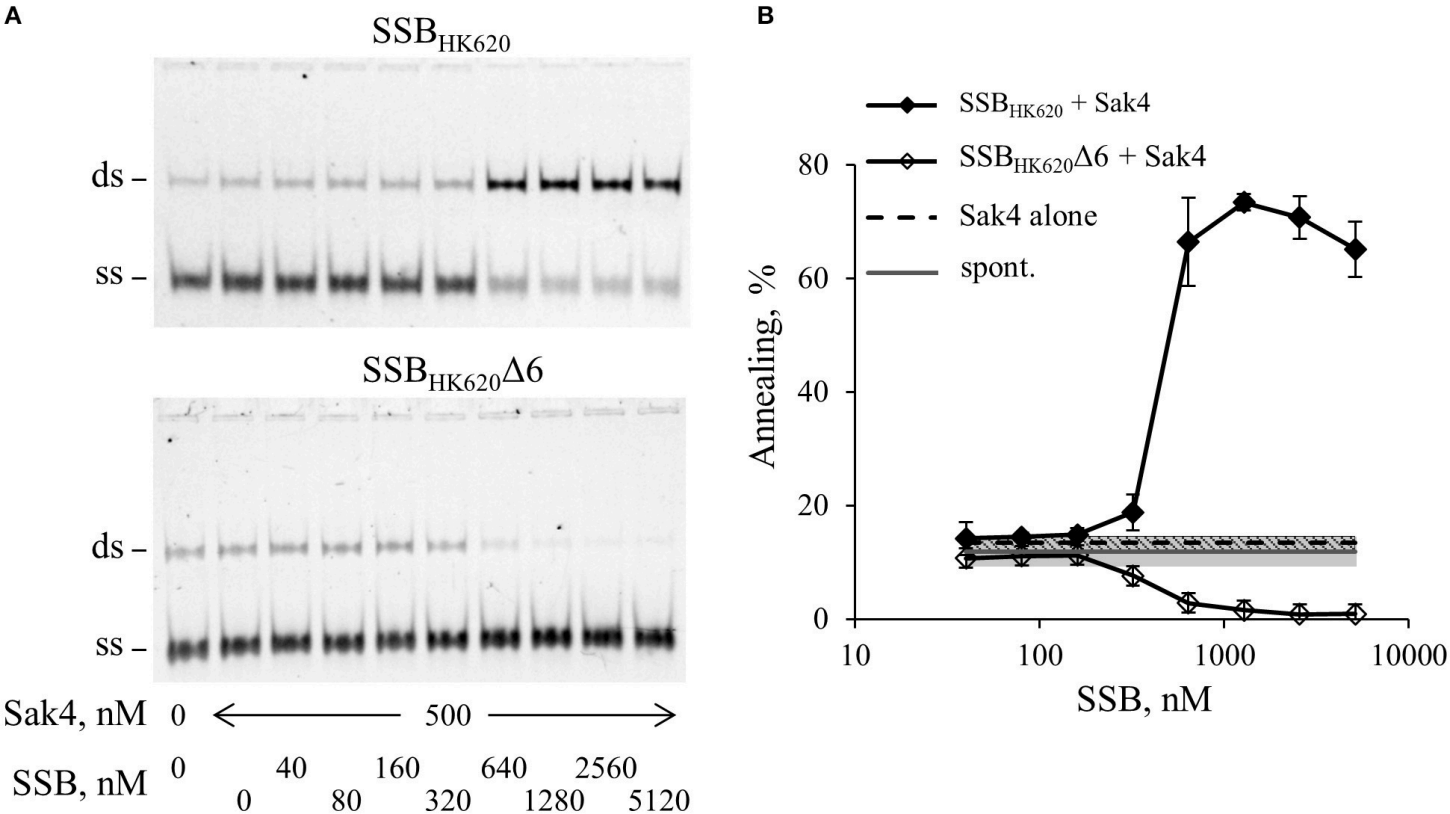
SSB_HK620_ stimulates the SSA activity of Sak4, depending on the presence of its C-terminal domain. **(A)** Effect of SSB_HK620_ and the truncated SSB_HK620_Δ6 proteins on the ability of Sak4 to promote SSA. GO47 (10 nM) was pre-incubated with variable concentrations of one of the SSB proteins (indicated above the gels) 15 min at 30°C and an additional 15 min with (500 nM) or without Sak4, as indicated. The SSA reaction was then started by the addition of the oligonucleotide GO34 (10 nM) for 30 min at 30°C. **(B)** Quantification of the SSA activity of Sak4 with SSB_HK620_ or SSB_HK620_Δ6, as indicated, from three independent experiments performed as described in **A**. Average and standard deviations are given. The gray line and gray band correspond respectively to the mean value and standard deviation of the spontaneous annealing (without proteins) in these experiments (spont.). The dashed black line and the hashed area indicate respectively the average and the standard deviation of the annealing performed by Sak4 (500 nM) without SSB proteins (Sak4 alone).

We then asked whether Sak4-mediated SSA was stimulated by its cognate SSB_HK620_. The assay was performed as described above, except that 0.5 μM of Sak4 was used instead of RecA. At this concentration, no SSA activity was noticeable when Sak4 was used alone (Figures 2B, 5). SSB_HK620_ led to a strong stimulation of SSA at concentrations above 640 nM (> 65% vs. 11.8% of spontaneous annealing, Figure 5B). Of interest, these concentrations of SSB_HK620_ corresponded to those favoring the C2-complex in the gel shift assay (Figure 4A). Such concentrations inhibited the RecA-mediated SSA (Figure 4B).

The last six residues of the SSB_HK620_ being mainly involved in the SSB_HK620_-dependent stimulation of the recombineering activity of Sak4 *in vivo* (Figure 3C), we assessed the role of this domain *in vitro*. An SSB_HK620_Δ6 protein deleted for these residues was purified, following the same steps as for the wild type protein. The elution volume from a SEC column of SSB_HK620_Δ6 was the same as it was for SSB_HK620_ (Supplementary Figure S5), and the binding of increased concentrations of SSB_HK620_Δ6 to the GO47 oligonucleotide led to results similar to those obtained with SSB_HK620_ (Supplementary Figure S6A). This strongly suggests that the truncated mutant SSB_HK620_Δ6 and the wild type protein share similar biochemical properties, alone or in the presence of ssDNA. However, SSB_HK620_Δ6 had an effect opposite to that of wild-type SSB_HK620_ when incubated with Sak4 for the annealing assay: rather than increasing the SSA activity of Sak4, it inhibited annealing altogether (Figure 5).

**Figure 6:**
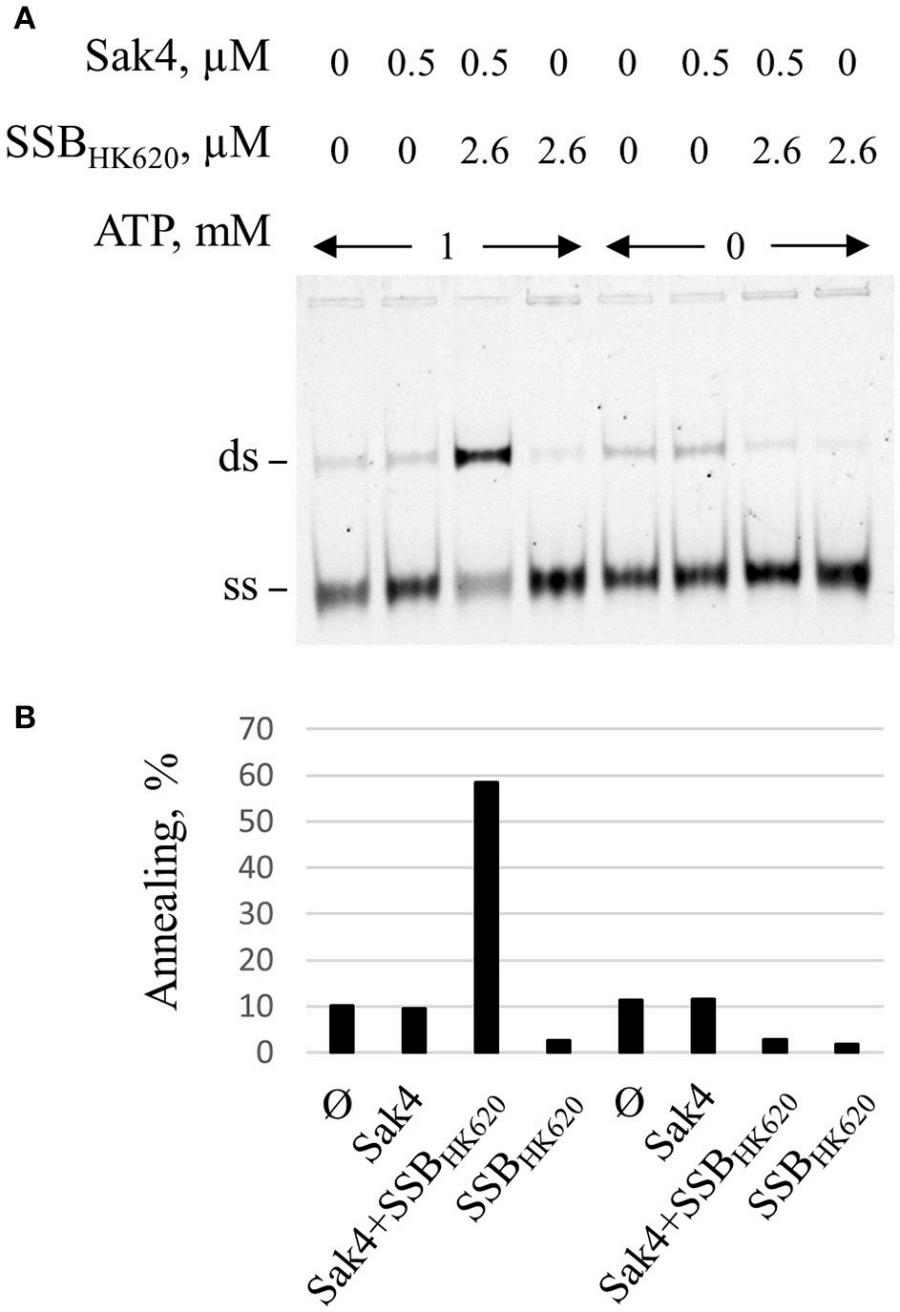
Stimulation of the Sak4-mediated SSA activity by SSB_HK620_ requires ATP. **(A)** GO47 (10 nM) was pre-incubated in the presence or not of 1 mM ATP, with or without 2.6 μM SSB_HK620_ for 15 min at 30°C and an additional 15 min with or without 0.5 μM Sak4, as indicated. The SSA reaction was then performed as described in the legend of Figure 5. **(B)** Quantification of the annealing shown in **A**.

Gp32 of phage T4 and eukaryotic RPA single-stranded binding proteins displayed annealing activities on certain kinds of DNA substrates and appropriate conditions (Kong et al., 1997; Bartos et al., 2008; Ramanagoudr-Bhojappa et al., 2014). We checked the SSA activity of the SSB_HK620_ and SSB_HK620_Δ6. Neither SSB_HK620_ nor the truncated mutant led to the formation of annealed products (Supplementary Figure S6B). In fact, they inhibited the spontaneous annealing of GO47 and GO34 at concentrations where C1 and C2 complexes occurred in the gel shift assay (compare Supplementary Figure S6A with Supplementary Figure S6B). Finally, the stimulation promoted by SSB_HK620_ was strictly ATP dependent (Figure 6). Knowing that (i) the SSA activity of Sak4 is ATP dependent (Figure 2B) and (ii) SSB_HK620_ alone did not display any SSA activity with or without ATP (Figure 6), we conclude that SSB_HK620_ stimulates the Sak4-mediated SSA *in vitro*. Its C-terminal domain is required for this process.

### Binding of Sak4 on SSB_HK620_-covered ssDNA requires the phage SSB C-terminal tail

**Figure 7:**
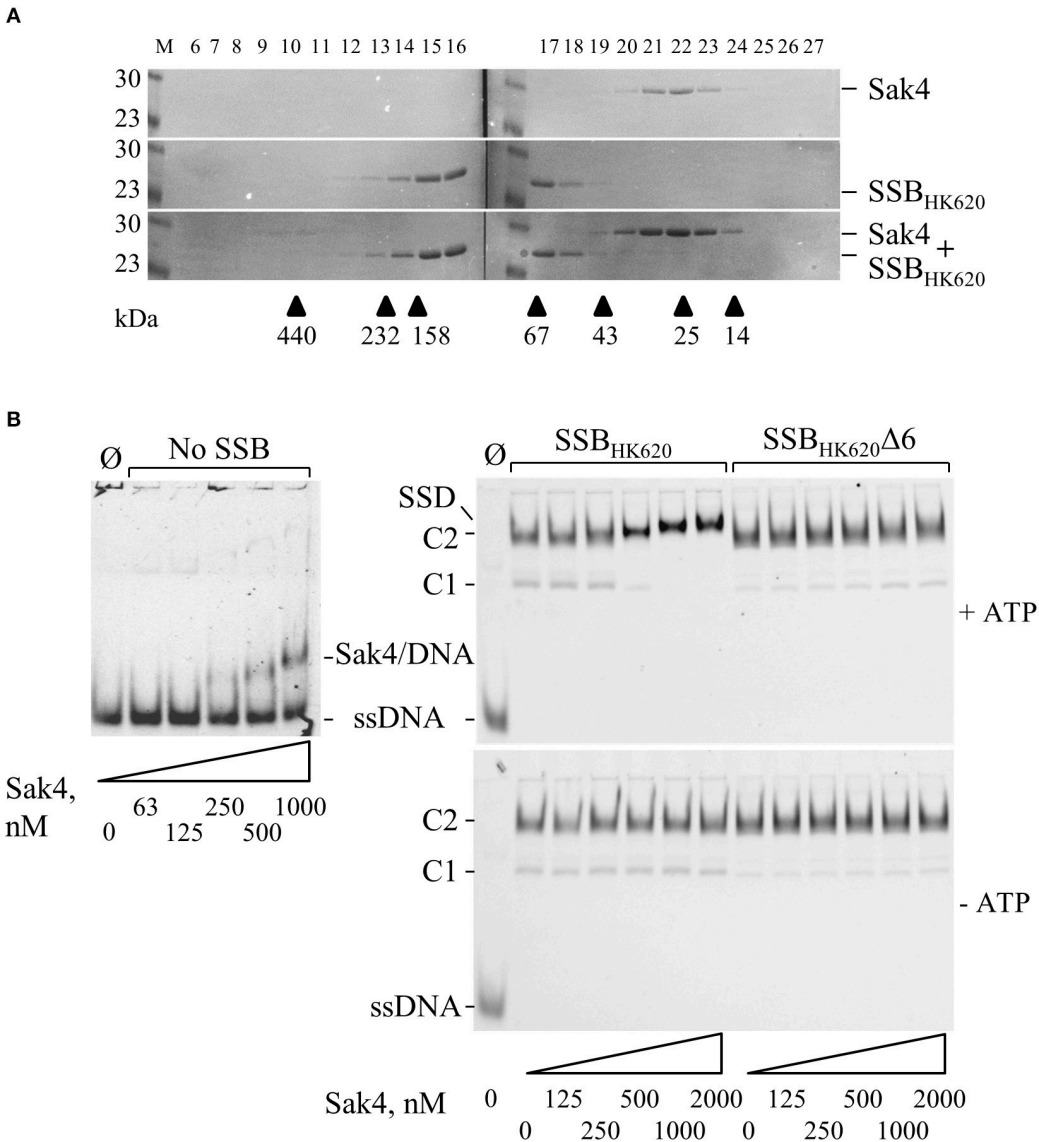
Interaction between Sak4 and SSB-covered ssDNA. **(A)** Sak4 and SSB_HK620_ do not interact in solution. The two phage proteins were loaded alone or together, as indicated on the right of the gels, on a gel filtration column and eluted fractions (indicated above the gels) were analyzed by SDS-PAGE 12.5%. Molecular weights (in kDa) of standard proteins used to calibrate the column (arrow heads) are indicated below the gel. M: molecular weight markers (in kDa). **(B)** The C-terminal tail of SSB_HK620_ and ATP are required for the binding of Sak4 to SSB-covered ssDNA. GO47 (10 nM) was pre-incubated 15 min at 30°C with (right panels) or without (left panel) SSB_HK620_ or the truncated mutant, as indicated above the gels. Then, Sak4 was added at the indicated concentrations for an additional 15 min before loading on a 5% acrylamide/bis-acrylamide (19:1) gel. Nucleoprotein complexes corresponding to one or two SSB (C1 or C2) or Sak4 (Sak4/DNA) bound to ssDNA and the ternary complex formed by SSB_HK620_, Sak4 and ssDNA (SSD) are indicated on the side of the gels. The band corresponding to GO47, without proteins added in the reaction (Ø), is indicated (ssDNA). Reactions performed with or without 1 mM ATP are shown in the upper gels and the lower gel, respectively.

Results described above strongly suggest a direct protein-protein interaction between Sak4 and its cognate SSB. Gel filtration experiments showed that the purified Sak4 was eluted with an apparent molecular weight of 29 kDa (Figure 7A), compatible with a monomeric status of the protein (theoretical molecular weight of 25.6 kDa). Mixed with SSB_HK620_ (95 kDa), an elution profile identical to each protein alone was observed (Figure 7A). Addition of ATP in the reaction and column buffer did not change the elution profile of the proteins (data not shown). Cross-linking experiments of these proteins alone with Sulfo-EGS confirmed the monomeric and oligomeric status of Sak4 and SSB_HK620_ in solution, respectively (Supplementary Figure S7). These stoichiometries were not changed in the presence of 1 mM of ATP. Migration patterns of these proteins alone or mixed together before addition of Sulfo-EGS were similar (Supplementary Figure S7). These results indicate that Sak4 and SSB_HK620_ do not interact directly in solution in our experimental conditions.

We next investigated whether the Sak4-SSB_HK620_ interaction required the prior formation of an SSB_HK620_-ssDNA complex, as suggested for the PriA-SSB interaction (Kozlov et al., 2010). SSB_HK620_-coated ssDNA complexes were formed at an SSB_HK620_ concentration of 5.1 μM (maximal stimulation of the Sak4 mediated SSA activity, Figure 5B). At this concentration, mostly C2 complexes were formed, although a slight amount of C1 was observed (Figure 7B, right panels). Sak4 was then added and incubated 15 min before analysis on gel. Remarkably, addition of 250 nM or higher concentrations of Sak4 led to the formation of a super-shifted band compared to the C2 complex in gel (annotated SSD for Sak4/SSB/DNA complex in Figure 7B, upper right gel). This SSD displayed a migration pattern different from C1 and C2 (same gel), but also from the Sak4/ssDNA complex (upper left gel). We conclude that SSD corresponds to a complex formed by Sak4, SSB_HK620_, and ssDNA. Of interest, 500 nM of Sak4 was sufficient to shift nearly all DNA bands corresponding to the C2 and C1 complexes, so the affinity of Sak4 for SSB_HK620_-ssDNA complex was at least four-fold higher than for uncoated ssDNA (<500 nM compared to ~1.85 μM in Figure 1B). This shows that SSB_HK620_-DNA complex stimulates/facilitates the binding of Sak4 onto ssDNA. Two parameters were required for the formation of SSD: (i) the presence of ATP in the reaction buffer (C1 and C2 complexes were not shifted without ATP, compare right gels in Figure 7B), and (ii) the C-terminal tail of the phage SSB (C1 and C2 complexes formed with SSB_HK620_Δ6 were not shifted after addition of Sak4 with or without ATP, compare right gels in Figure 7B).

### Sak4 does not perform strand exchange, nor stimulate the RecA-mediated reaction *in vitro*, whereas SSB_HK620_ stimulates the RecA-mediated strand exchange reaction

**Figure 8:**
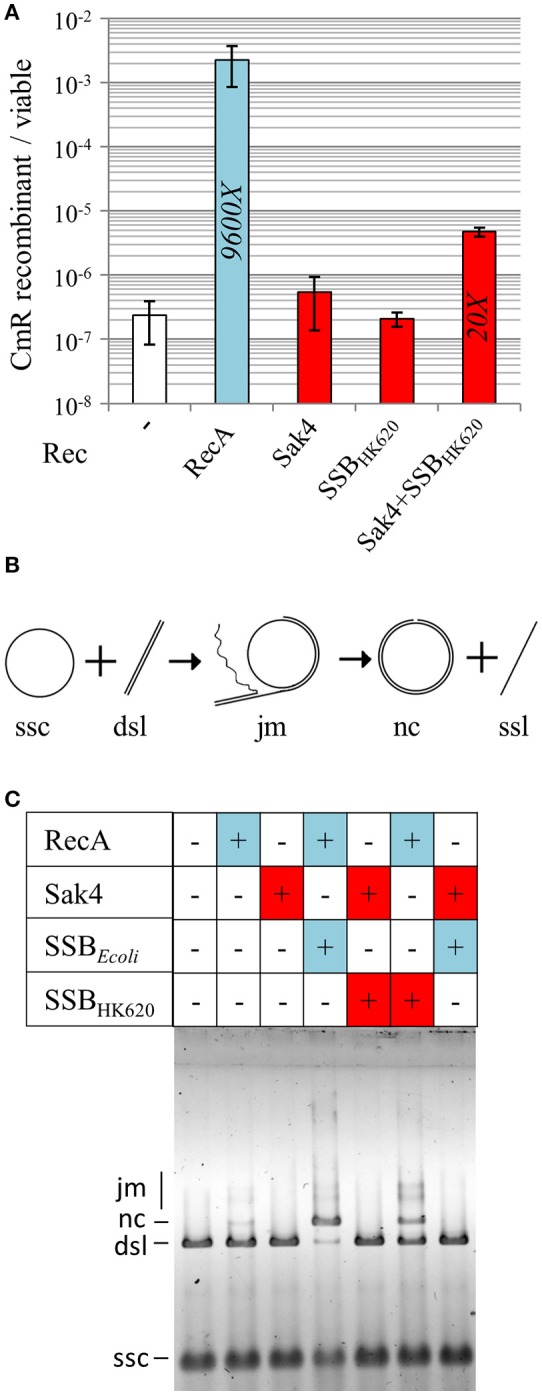
Sak4 and SSB promote a low level of cross-over recombination events *in vivo* but no strand exchange *in vitro*. **(A)** Recombination efficiency of RecA and Sak4 (“Rec” legend) in a RecA^−^ background. This recombination efficiency was determined by calculating the ratio of CmR ex-conjugants/viable recipient cells after conjugation (see section Materials and Methods). Recombination efficiency of the RecA^−^ strain expressing *sak4* alone (MAC2142), *sak4* and *ssb*_HK620_ together (MAC2081) or *ssb*_HK620_ alone (MAC2140) are shown as red bars and compared to the recombination efficiency of the RecA^−^ strain expressing *recA* (MAC 2080, blue bar). Experiments were performed three times and error bars indicate the standard deviation around the mean. The fold increases, relative to the spontaneous recombination obtained in the RecA^−^ background without expression plasmid (strain MAC1494, white bar), is indicated in each bar, only if the recombination efficiency is statistically different from the background level (*p* < 5%, Student *t*-test). **(B)** Scheme of the strand exchange reaction: circular ssDNA (ssc) bound to the recombinase reacts with its homologous linear dsDNA (dsl) to form joint molecules (jm) between ssc and dsl. The final products of the strand exchange are a nicked circular dsDNA (nc) and linear ssDNA (ssl). **(C)** Recombinases (1.85 μM, +: presence, –: absence) were pre-incubated with the ssc substrate (10 μM in nucleotides) and a SSB protein (0.5 μM, +: presence, –: absence) was added for a second pre-incubation. The dsl substrate (10 μM in nucleotides) was then added. Reactions were analyzed after 30 min at 37°C on a 0.8% agarose gel.

Sak4 being similar to the central domain of RecA, we asked whether Sak4 could complete cross-over recombination *in vivo*, in a recombination conjugation assay. For this, a CmR suicide plasmid pJA3 (Amarir-Bouhram et al., 2011) was used, which depends for its replication and conjugation on genes introduced into the chromosome of the donor strain MFD*pir* (Ferrières et al., 2010). Upon conjugation with a recipient strain devoid of the *pir* plasmid replication protein, the only way for the *cat* gene to be maintained is to recombine by single or double cross-over with the *lacZ* gene of the recipient, as pJA3 contains two 1 kb regions spanning the 5′ and 3′ end of *lacZ* and flanking the *cat* gene (Supplementary Figure S8). Using a *recA* recipient strain, a background level of 2 × 10^−7^ CmR/recipient cells was obtained (Figure 8A). When plasmid pJ192 (expressing *recA*) was added to the recipient strain, ~10^4^-fold more CmR ex-conjugants were obtained. Interestingly, expression of the *sak4*-*ssb*_*HK*620_ construct from pGH19 in the recipient strain led to approximately 4.7 × 10^−6^ CmR/recipient cells, 20-fold more than the background level. In the same conditions, expression of *sak4* or *ssb*_*HK*620_ alone led to not statistically different recombination frequencies compared to the control. This result highlights some modest *in vivo* recombination activity dependent on the Sak4/SSB_HK620_ duet.

The cross-over recombination mediated by RecA is dependent on its strand exchange activity (SE). We then asked whether Sak4 could display such SE activity. The classical assay which follows the exchange of a strand from the linear ΦX174 dsDNA (dsl) on a homologous circular ssDNA was used (ssc, Figure 8B). Both RecA and Sak4 were tested, either with SSB_*Ecoli*_, SSB_HK620_, or alone (see Materials and Methods). RecA at 1.85 μM generated traces of joint molecules intermediates (jm) and nicked circular dsDNA molecules (nc, one of the two products of the reaction) after 30 min of reaction. The presence of SSB_*Ecoli*_ (0.5 μM) permitted an almost complete SE reaction, i.e., nearly all the ΦX174 dsDNA available was replaced by nicked circular dsDNA molecules (Figure 8C, compare lanes 2 and 4), as expected (Kowalczykowski and Krupp, 1987). However, for Sak4 (1.85 μM) no indication of strand exchange activity was observed (no jm nor nc products observed, Figure 8C, lane 3), even in the presence of SSB_HK620_ or SSB_*Ecoli*_ (0.5 μM, lanes 5 and 7, respectively). Even at 150 mM of NaCl, the best condition for measuring Sak4 SSA activity, Sak4 did not perform SE (not shown). Interestingly, SSB_HK620_ (0.5 μM) also stimulated the RecA-dependent reaction, albeit to a lesser extent compared to SSB_*Ecoli*_ (compare lanes 4 and 6).

The *E. coli* RecA paralog Sms stimulates RecA-mediated strand exchange (Cooper and Lovett, 2016). Sak4 does not share the characteristics of Sms, namely an N-terminal Zinc-finger motif, a C-terminal S5 domain 2, and a KNRFG motif signature. We nevertheless tested the effect of Sak4 on the SE reaction mediated by RecA. In order to detect positive or negative influence of Sak4, the reaction was stopped half-way, at 12 min. Neither stimulation nor inhibition of SE activity by Sak4 was observed (Supplementary Figure S9A). Reactions were repeated with varying order of additions, and did not reveal any effect of Sak4 on RecA (Supplementary Figure S9B). We conclude that Sak4 has no effect on SE reactions, either alone or as a RecA companion.

## Discussion

The present biochemical and genetic characterization of Sak4 leads to the straightforward conclusion that it is an SSAP needing its cognate SSB for full activity *in vivo* and *in vitro*. This constitutes the first report of a stimulatory effect of an SSB for the annealing activity of a phage SSAP. Based on these observations, we propose that Sak4 are RecA-core only proteins that could represent a functional link between two distinct types of recombinases, i.e. SSAPs that are recombination mediator proteins, able to stimulate strand exchange proteins, and strand exchange proteins themselves. We discuss below the characteristics of Sak4 that sustain this proposal.

### Sak4 is an ATP dependent SSAP able to anneal complementary ssDNA covered by its cognate SSB

Sak4 has a dual character: on the one side, it behaves like an SSAP. First, it promotes recombineering *in vivo*, to levels comparable to the well-characterized Rad52-like Redβ SSAP, depending on the locus targeted, and provided its companion SSB is co-expressed. RecA is unable to do so (even when some phage SSB is present, not shown). Second, the purified protein has an SSA activity *in vitro* that is stimulated by the presence of SSB_HK620_ bound to ssDNA. Only few documented SSAPs are able to anneal SSB-covered complementary DNA molecules, i.e., eukaryotic Rad52, bacterial RecO, and T4 phage UvsY (for review see Kowalczykowski, 2015). These SSAPs are recombination mediator proteins that stimulate DNA strand exchange proteins (Rad51, RecA, UvsX, respectively) when their cognate SSBs are present (RPA, SSB, and Gp32, respectively). However, we show here that Sak4 does not influence the strand exchange activity of *E. coli* RecA. Since phage HK620 does not contain any gene coding for another known strand exchange protein (Lopes et al., 2010), these results suggest that Sak4, while displaying some functional homologies with Rad52, RecO, and UvsY, is not a recombination mediator protein.

On the other side, Sak4 is a RecA-core only protein lacking the C-terminal domain of RecA (Lopes et al., 2010). This makes it profoundly distinct from SSAP such as Rad52, RecO and UvsY which do not share any homology with RecA. In line with the presence of Walker A and B motifs in Sak4, and in contrast to other SSAPs, we show here that DNA binding and SSA activities are ATP-dependent. ATP may promote some structural changes in Sak4, releasing its DNA binding site, otherwise embedded inside the folded protein. Structural studies of Sak4 in the absence or presence of ATP are required to test this hypothesis.

We reported earlier that recombineering by the Sak4 of phage PA73 was 4-fold higher than background (Lopes et al., 2010). A mutation of its Walker A box (K30A) diminished recombineering slightly (by 1.7-fold), but the mutant still maintained an activity 2-fold above background. This led us to surmise that the ATP binding site was dispensable for recombineering (Lopes et al., 2010). However, since the *ssb* gene of PA73 proved toxic in *E. coli*, we could not confirm this result with much higher levels of recombineering. In view of the present results showing that for phage HK620, Sak4 is strongly stimulated by its cognate SSB, and needs ATP for its activity, we now hypothesize that the ATP binding motifs of phage PA73 Sak4 are also required.

Sak4 differs from RecA in its requirement for ATP. Indeed, the binding of RecA to ssDNA is ATP-stimulated but RecA is able to bind DNA and to promote SSA without ATP (Bryant and Lehman, 1985; Bryant et al., 1985). The C-terminal domain of RecA, that is thought to comprise the second strand DNA-binding site (Aihara et al., 1997; Yang et al., 2015), is absent from Sak4 and could be responsible for this difference. It has to be noticed that the RecA-homolog UvsX from phage T4 requires ATP i) to bind Gp32-covered ssDNA in certain conditions without the need for its recombination mediator UvsY (Liu et al., 2013) and ii) to bind certain kinds of ssDNA not covered with Gp32 (Maher and Morrical, 2013). We therefore propose that the ATP dependent binding and annealing of Sak4 are reminiscent of properties shared by strand exchange proteins in specific conditions.

In the present study, we compared the annealing efficiency of a set of phage and bacterial recombination proteins. Among the four proteins tested, three shared the core domain of RecA, namely Sak4, UvsX, and RecA, but only Sak4 performs recombineering *in vivo*. The additional DNA-binding domains of UvsX and RecA may specialize these proteins toward strand exchange, instead of recombineering, *in vivo*. Interestingly, we observed that co-expression of *sak4* with its cognate *ssb* was able to promote post-conjugation recombination events at a 20-fold higher frequency compared to the spontaneous recombination events (but 500-fold lower than the one mediated by RecA, Figure 8A). Further work is needed to decipher the Sak4-dependent mechanism allowing integration of a plasmid displaying sequence homology with the chromosome *in vivo*. Somehow, the SSB companion of Sak4 can be viewed as bringing a second DNA site to the protein, and thereby promote some modest level of cross-over recombination *in vivo*.

### An ssDNA dependent interaction between SSB_HK620_ and Sak4 may increase the annealing activity of Sak4

Sak4 needs the presence of its cognate SSB protein *in vivo* to carry out fully efficient ssDNA recombineering, and this is the first time that an SSB is reported to be needed for this reaction. This is also true for the annealing activity of Sak4 *in vitro* on two complementary 81-mer ssDNA molecules. Both *in vivo* and *in vitro*, this SSB_HK620_-dependent stimulation requires the six C-terminal amino acids of the protein, which display homology with the acidic C-terminal tail of several bacterial and phage SSBs. Because this tail is required for interaction between bacterial or phage SSBs with several genome maintenance proteins from bacteria (Shereda et al., 2008) or phages (Kong and Richardson, 1998), we hypothesize that SSB_HK620_ interacts with Sak4, thereby facilitating its recruitment to the ssDNA and stimulating its annealing activity. Surprisingly, we were able to demonstrate this interaction only if SSB_HK620_ was bound to ssDNA. Replacing the wild-type protein by the truncated mutant SSB_HK620_Δ6 on ssDNA abolished this interaction. This result shows that the C-terminal tail of the phage SSB, once bound to ssDNA, mediates Sak4 binding. In contrast, SSB_HK620_Δ6 acts as a barrier against the binding of Sak4 onto ssDNA. This suggests that the length of free ssDNA in a C2 complex (containing two tetrameric SSB_HK620_) is insufficient to allow the direct binding of Sak4 and reinforces our proposal of a direct physical interaction between Sak4 and its cognate SSB.

Interestingly, eukaryotic Rad52 interacts with its cognate RPA and this interaction stimulates the SSA activity of yeast Rad52 (Park et al., 1996; Shinohara et al., 1998; Sugiyama et al., 1998). Bacterial RecO proteins also interact with SSB and this interaction facilitates, or at least does not inhibit, the RecO-mediated SSA activity (Shereda et al., 2008; Costes et al., 2010; Manfredi et al., 2010). The C-terminal domain of the bacterial SSB is required for this interaction and influences the RecO mediated SSA activity (Ryzhikov et al., 2011). Finally, the T4 UvsY, that helps the strand exchange protein UvsX, interacts with Gp32 and this interaction is also dependent on the C-terminal tail of this SSB homolog. Therefore, all three recombination mediator proteins have conserved this property to interact with SSB.

Why then Sak4 and SSB_HK620_ should not interact in the absence of ssDNA? Presumably, the SSBHK620 C-terminal tail is not accessible for Sak4 in the absence of ssDNA. Upon ssDNA binding, SSB_HK620_ could release its C-terminal tail, and make it available for Sak4. The acidic C-terminal tail of SSB_*Ecoli*_ was reported to interact weakly with its DNA binding site (Su et al., 2014), and the affinity of SSB_*Ecoli*_ for its partners PriA and the DNA polymerase Chi subunit is increased when bound to ssDNA (Kozlov et al., 2010). Similarly, a recent interaction between RecA and SSB coated on ssDNA has been proposed (Wu et al., 2017), while no evidence for a direct interaction between these proteins has been described in the absence of DNA (Shereda et al., 2008). Sak4 sharing homology with RecA, this recent observation reinforces our proposal that Sak4 interacts directly with SSB_HK620_ on DNA. How Sak4 displaces SSB_HK620_ from ssDNA, if required, and why should an SSB_HK620_ concentration allowing formation of the C1 complex not stimulate SSA, remains unresolved at present, and structural studies are needed to answer these questions.

### Comparing Sak4 functions with those of RAD51/RecA paralogs

Based on its sequence, Sak4 resembles RAD51 paralogs (Lopes et al., 2010). These proteins are the field of intense research activities in the recent years, as they appear to modulate the fate of the recombination process.

In fungi, the Rad55-57 proteins, and the Shu complex, both made of Rad51 paralogs, have roles narrowly connected with the Rad51 filament, which they strengthen against the anti-Rad51 activity of Srs2 (Bernstein et al., 2011; Liu et al., 2011). We could not detect any indication of a functional interaction between Sak4 and RecA_*Ecoli*_, neither *in vivo* nor *in vitro*. Indeed, Sak4-mediated recombineering occurred at similar levels in RecA^+^ or *recA*-deleted *E. coli* strains.

Sak4 is also distinct from the *E. coli* Sms protein, which acts just afterwards RecA, during the branch migration step. Both genetics and biochemical studies show that Sms has a positive role on the homologous recombination process, by pushing forward the Holliday junction intermediate (Beam et al., 2002; Cooper et al., 2015; Cooper and Lovett, 2016). For Sak4, no effect on the yield of RecA-mediated strand exchange reaction *in vitro* has been observed, at least in our assay.

Structurally, Sak4 is most similar to the archaeal RadB protein (Lopes et al., 2010). However, it does not share many biochemical features with it. The Sak4 binding to ssDNA is strictly ATP-dependent, whereas RadB binds ssDNA as well in the presence or absence of ATP (Komori et al., 2000). Furthermore, in contrast to Sak4, RadB inhibits the RadA-dependent strand exchange reaction (Komori et al., 2000). The other Rad51 paralog aRadC is also difficult to place in line with Sak4. It binds efficiently ssDNA in the absence of ATP, and its effect on RadA-mediated D-loop formation is again inhibitory (McRobbie et al., 2009).

Among all RecA/Rad51 paralogs, it seems therefore that Sak4 exhibits activities more similar to the RAD51BCD-XRCC2 complex, which in the case of humans, promotes SSA *in vitro* (Yokoyama et al., 2004), and in the case of plants, permits SSA *in vivo* (Serra et al., 2013). Our observation of the key role of SSB in Sak4 activity differs from the known properties of BCDX2 complexes, and suggests future avenues of research.

## Materials and methods

### Distant homology searches on SSB

Distant homologs of HkaK from phage HK620 (gi:13559834, Q9AZ30_BPHK6, named hereafter SSB_HK620_) were searched using Phagonaute (Delattre et al., 2016), with an HHsearch confidence probability cut-off of 95%. SSB_HK620_ was found to be related to the Gp45 protein of the coliphage N4 (Supplementary Figure S3), which is an atypical single-stranded DNA binding protein without homology with bacterial SSB proteins (Lindberg et al., 1989; Choi et al., 1995). Moreover, two proteins annotated as SSB were retrieved, from phages Lc-Nu and PhiAT3 (Supplementary Figure S3). To ascertain that proteins annotated as SSB produced by phages Lc-Nu and PhiAT3 were homologous to experimentally proven SSB, these two proteins were compared to the PDB70 database available at http://toolkit.lmb.uni-muenchen.de/ using HHpred. Each of them identified as best hit the SSB of *E. coli* (probability above 99%, with 14 and 17% sequence identity). However, SSB_HK620_ did not match directly with SSB of *E. coli* with HHpred.

### Strains and oligonucleotides

*E. coli* strains used in this study are listed in Supplementary Table S1. Oligonucleotides for recombineering and *in vitro* SSA tests are described in Supplementary Table S2, while those for plasmid constructions are in Supplementary Table S3.

The MAC1802 strain is deleted for its *mutS* and *recA* genes, and contains in addition a mutated *cat* gene (*catss*) that was constructed as follows. Site directed mutagenesis was performed using the two complementary oligonucleotides J85 and J86 (Supplementary Table S3), to introduce two stop codons separated by 6 nucleotides into the *cat* gene of pACYC184 (Chang and Cohen, 1978). The two stop codons generate G:A and T:G mispairs during the recombineering experiment using oligonucleotide Maj98, which has the polarity of a lagging strand. Oligonucleotides J84 and J87 flank the *cat* gene of pACYC184 and overlap two *BmgBI* sites of this plasmid. The two halves of the mutated *cat* gene were amplified with the J84-85 and J86-87 pairs (652 and 243 bp fragments, respectively), and the full *catss* mutated gene was then produced using these two PCR fragments as initial primers, as well J84 and J87. The full gene fragment was then cloned into the *BmgBI* site of pKD4 (Datsenko and Wanner, 2000). This *catss* mutated gene, together with the KanR marker of pKD4 were next introduced by recombineering into the *thi* operon (10 kbp away from *rpoB*), in place of *thiE* and *thiF*, and in the same orientation, of strain JJC40 (AB1157 *hsdR*), using oligonucleotides Maj80 and Maj81. The resulting cassette was next transduced by P1 into AB1157 (strain MAC1716), with KanR selection. The *mutS*Ω (specR) allele (Brégeon et al., 1999) was next introduced by P1 transduction in this strain (MAC1774), and finally the Δ*recA*306 *srl*:*Tn*10 allele was added by P1 transduction (from strain GY5902, a gift from Dr. S. Sommer), to give strain MAC1802.

All plasmid constructions are described in Supplementary Table S4. Except pOS10, plasmids (column 1) were constructed by the cloning of a PCR product (columns 3 to 6) into a plasmid (column 7 and 8). PCR were performed with the high-fidelity polymerase Phusion (Roche), using the DNA matrix indicated in the fourth column, and the oligonucleotides indicated in columns 5 and 6. The vector (column 7) and the PCR product were digested by the enzyme stipulated in column 8. Integrity of the PCR insert was verified by sequencing. pOS10 was constructed by replacing the *NdeI*-*StyI* fragment of the pGH19 containing the 3′ part of the *ssb*_HK620_ by the *NdeI*-*StyI* fragment of the pOS8 containing the 3′ part of *the ssb*_HK620_Δ6 mutant.

### Recombineering assay at the *catss* locus

Recombineering was performed to revert the *catss* allele (strain MAC1802) into the wild-type allele, with oligonucleotide Maj98, or a series of derivatives with increased divergence to the *catss* template (see Supplementary Table S2). A culture of the bacterial strain to be tested was grown over-night at 30°C in LB supplemented with 100 μg/mL ampicillin, diluted 100-fold in 50 mL of the same medium, and grown at 30°C until an OD_600nm_ of 0.2. The genes placed under the Para promoter were induced by addition of L-arabinose to a final concentration of 0.2% and the culture was then shifted to 37°C. After 45 min (OD_600nm_ between 0.6 and 1), cells were pelleted in a pre-cooled centrifuge (4°C) for 7 min at 5,200 g, resuspended in 50 mL of ice-cold glycerol 10% and centrifuged again (same parameters). The pellet was washed again twice, with 1 mL of cold glycerol 10% (centrifugation 1 min, 7,500 g), resuspended in 300 μL of cold glycerol 10% and used directly. In an earlier version of the protocol (Lopes et al., 2010 and Supplementary Figure S4), frozen competent cells were used. This diminished by 10-fold cell viability, but recombineering efficiencies (ratio of recombinants to viable cells) remained comparable with the non-frozen cells protocol. Electroporation was always performed with 1 μg of oligonucleotide, gently mixed with 100 μL of competent cells and electroporated at 2.5 kV, with a resistance of 200 Ω and a capacitance of 25 μF. Cells were diluted in 900 μL of LB and grown for 1 h at 37°C.

Selection was done in soft agar, as follows. Dilutions of cells were mixed to 5 mL of molten Top Agar 7 (10 g/L Bactotryptone, 2.5 g/L NaCl, and 7 g/L Agar) and plated on LB agar to count total viable cells. To select the CmR recombinants, the same procedure was used, except that after 2 h of growth in the top-agar layer at 37°C, a second top-layer of a mix of 5 mL of molten Top Agar 7 supplemented with 140 μg/mL chloramphenicol was added, for a final concentration of 20 μg/mL in plate. Bacteria were numerated after 24 h of growth at 37°C. The frequency of recombination was estimated by the number of recombinant cells divided by viable cells (average and standard deviation of at least 3 repeats).

### Recombineering assay at loci other than *catss* and with various expression vectors

Recombineering at *catss* was compared with two other loci of the *E. coli* chromosome, usually in *recA*-deleted backgrounds: (i) at the *rpoB* gene, with oligonucleotide Maj106, and selection on rifampicin. The advantage of this assay is its portability, because the recipient gene is not mutated, and the C:C mismatch created by pairing Maj106 to *rpoB* is resistant to mismatch repair. However, a high background of spontaneous annealing is observed at this locus (Supplementary Table S6). (ii) At the *galK* locus with oligonucleotide 144 (Costantino and Court, 2003), in strain G205, a *recA* derivative of HME57 (Datta et al., 2006, Supplementary Table S1), for comparison purposes with published data using this locus. Recombineering at the *rpoB* locus was measured as described above for the *catss* locus except that the selection was done using a second top-layer of a mix of 5 mL of molten Top Agar 7 supplemented with 350 μg/mL rifampicin, for a final concentration of 50 μg/mL in plate. For the Gal^+^ recombination assay, M63 minimal medium plates supplemented with 2 mM MgSO4 and biotin 0.001% were used, in which 0.2% glucose was added for the viable count, or 0.2% galactose for the count of Gal+ recombinants.

In addition, to compare recombinase expression levels, two plasmid expression systems were used: (i) one based on a Para promoter, described above, (ii) another based on the strong P_T7_ promoter, derived from plasmid pJ411 (Menlo Park, CA), with medium copy number (some of these constructions were used also for protein purification). For this second assay, strain ER2566 or its derivatives were used (Supplementary Table S1), in which the T7 polymerase gene is placed under the IPTG-inducible Plac promoter into the chromosome. For strains containing these plasmids, cultures were done in LB supplemented with 50 μg/mL kanamycin and induction of genes placed under the P_T7_ promoter was done by addition of 100 μM IPTG. All results are reported in Supplementary Table S6.

### Conjugations

Conjugations were performed between the MAC1628 donor strain containing the pJA3 plasmid (Amarir-Bouhram et al., 2011) and various recipient strains corresponding to MG1655 *recA* mutant strains containing L-arabinose inducible genes on pKD46 derivative plasmids (see Supplementary Tables S1, S4). Expression of these genes was induced by adding 0.2% of L-arabinose in exponentially growing cultures (OD_600nm_ = 0.1) of the recipient strains in LB medium at 30°C for 1 h. Recipient cells (1 mL, OD_600nm_ = 0.4) were then mixed with exponentially growing culture of the donor strain (OD_600nm_ = 0.4) using a ratio of 2 recipient cells per donor cell. Conjugations were done on filters at 30°C for 2 h. After conjugation, filters were taken and cells resuspended by vortexing in LB. Viable recipient cells were determined by plating various dilutions of the mixtures on LB rich medium devoid of diaminopimelate, to counter-select the donor cells. CmR recombinants were detected on LB plates supplemented with 20 μg/mL of chloramphenicol, Colonies were numbered after 24 h of incubation at 37°C.

### Protein purifications and purchased proteins

#### Sak4 purification

Sak4 was purified from *E. coli* ER2566 transformed by pSMG288. Cells were cultured in 400 mL of LB supplemented with 30 μg/mL of kanamycin at 37°C until an OD_600nm_ of 0.6. Gene expression was induced at 30°C for 3 h by addition of IPTG (100 μM final concentration) to the culture. Cells were then harvested by centrifugation; pellets were resuspended at 4°C in 20 mL of buffer A1 (100 mM Tris-HCl pH 8.5, 5 mM MgCl_2_, 1 mM DTT, 10% glycerol, 1% triton X100) and frozen in liquid nitrogen before storage at −80°C until purification.

After thawing, cell solution was incubated with 2.5 mg/mL of lysosyme on ice for 15 min before sonication. All subsequent steps were performed at 4°C and centrifugations were done for 20 min at 20,000 g. The lysate was centrifuged and 0.5% of polyethyleneimine (PEI) was added to the supernatant. After 5 min of incubation, the solution was centrifuged and the supernatant containing Sak4 was supplemented with ammonium sulfate (AS) up to 45% of the saturation concentration and incubated for 15 min. Precipitated proteins were discarded by centrifugation. Sak4 was present in the supernatant at this step and was precipitated by adding AS at 65% of the saturation concentration. Precipitated proteins were separated from the soluble fraction by centrifugation and resuspended in 45 mL of buffer B1 (50 mM Tris-HCl pH 8, 1 mM DTT, 5% glycerol). After filtration through a 0.22 μM filter, proteins were loaded on a 5 mL HiTrap Heparin column equilibrated in buffer B1 and eluted with a 75 mL linear gradient from 50 mM to 500 mM of NaCl in buffer B1. Fractions containing the purified Sak4 were pooled and proteins were precipitated by addition of AS up to 65% of saturation. After centrifugation, the pellet was resuspended in 0.5 mL of buffer B1 supplemented with 150 mM NaCl, centrifuged, and the supernatant was loaded on a Superdex 200 size-exclusion column equilibrated in the same buffer. Eluted fractions containing Sak4 were pooled and glycerol was added to a final concentration of 50% prior storage at −20°C.

#### SSB purifications

Wild-type HkaK protein from phage HK620 (named hereafter SSB_HK620_) was produced untagged in strain ER2566 transformed with plasmid pSMG279, as we noticed that even a 3-residues long N-terminal scar was sufficient to decrease by 100-fold its affinity for ssDNA. Bacteria were grown at 30°C in 500 mL of LB medium supplemented with 30 μg/mL kanamycin to OD_600nm_ = 1.0. Production of protein was induced with 0.5 mM IPTG for 3 h at 30°C. Cells were harvested by centrifugation and pellet was resuspended in 25 mL of buffer A2 (50 mM Tris-HCl pH 8). Cells were broken by incubation with 2.5 mg/mL of lysosyme on ice for 15 min before sonication. The lysate was centrifuged at 20,000 g for 20 min at 4°C. All subsequent steps were carried out at 4°C. Two mL of PEI 10 %, pH 8 were added to the lysate supernatant to precipitate SSB_HK620_ with the nucleic acids; the mixture was stirred for 5 min and then centrifuged 5 min at 5,000 g. To solubilize back SSB, the pellet was resuspended in 25 mL of buffer A2 supplemented with 200 mM NaCl, centrifuged 5 min at 5,000 g, and the supernatant was kept. The SSB_HK620_ contained in this fraction was then precipitated by addition of AS up to 30% of saturation. After centrifugation, the pellet was resuspended in 10 mL of buffer A2. Proteins were loaded on a 5 mL HiTrap Heparin column equilibrated in buffer A2 and eluted with a 75 mL linear gradient from 0 to 500 mM of NaCl in buffer A2. Fractions containing the purified SSB_HK620_ were pooled and dialyzed against buffer B2 (50 mM Tris-HCl pH 8, 0.4 M NaCl, 50% glycerol) prior to storage at −20°C.

The truncated mutant SSB_HK620_Δ6 was produced in strain ER2566 transformed with plasmid pOS8 and purified essentially as described for the wild type SSB_HK620_ except that the concentration of AS used for its precipitation was 35%.

#### Purification of UvsX and Redβ

UvsX from phage T4 and Redβ from phage λ were produced with an N-terminal His-GST tag, from Lemo21-DE3 *E. coli* transformed by pETM30-derived plasmids (Supplementary Table S4). Cells were cultured in 800 mL of LB supplemented with kanamycin (30 μg/mL) at 37°C until an OD_600nm_ of 1. The culture was then transferred at 20°C and the recombinases tagged by GST were induced by IPTG at a final concentration of 100 μM. Cells were then cultured overnight at 20°C and harvested by centrifugation at 5,000 g for 30 min at 4°C. They were resuspended at 4°C in buffer A3 (100 mM Tris-HCl pH 7.5, 100 mM NaCl, 5 mM MgCl_2_, 1 mM DTT, 10% glycerol, 1% triton X100, 1 mM PMSF, 8 μg/mL aprotinine). These solutions were supplemented by 2.5 mg/mL of lysosyme and stirred on ice for 15 min. Cells were then lysed by sonication. The lysate was clarified by centrifugation (30 min at 30,000 g at 4°C) and mixed with 5 mL of GSH-agarose beads (Sigma) resuspended in buffer B3 (10 mM Tris-HCl pH 7.5, 5 mM MgCl_2_, 100 mM NaCl). The mixture was incubated at 4°C for 1 h under agitation. The beads were then transferred on a gravity flow column (Biorad) and washed with 25 mL buffer B4 (10 mM Tris-HCl pH 7.5, 5 mM MgCl_2_, 1 M NaCl). A second wash was done with 25 mL of buffer B3. The fusion proteins were eluted with 15 mL of a buffer B5 (10 mM Tris-HCl pH 7.5, 5 mM MgCl_2_, 150 mM NaCl, 1 mM DTT, and 10 mM GSH). The his-GST-tag was finally removed by the addition of the his-tagged TEV protease at a concentration of 1/100 (w/w) overnight at 4°C. Ten mM final concentration of imidazole (Sigma) was added to the solution to prevent unspecific binding to the column and the his-GST-tag and his-TEV protease where then removed using a 5 mL His-trap fast flow column (GE Healthcare). The flow through containing the proteins was dialyzed overnight at 4°C against buffer B3 supplemented with 1 mM DTT. UvsX and Redβ proteins were frozen as small aliquots in liquid nitrogen and stored at −80°C.

The purified RecA and SSB (hereafter SSB_*Ecoli*_) proteins of *E. coli* were purchased from Epicentre (Tebu-bio). The GST was purified as described previously (McGovern et al., 2016).

The concentration of all purified proteins, except SSB proteins, used in this study was determined by the OD_280nm_ and the ϵ of each protein. Concentration of SSB proteins was determined by a Bradford Protein Assay (Bio-Rad). SDS-PAGE of the purified proteins is shown in Supplementary Figure S1. Absence of visible DNA degradation at the end of the different assays performed in this study indicates that the purified proteins were devoid of nuclease activities.

### Gel shift assay

Unless otherwise stated, gel shift experiments were done in a final volume of 20 μL of a reaction buffer SSA (13 mM Tris-HCl pH 7.5, 5 mM MgCl_2_, 169 mM NaCl, 0.1 mg/mL BSA, and 1 mM DTT) containing or not 1 mM ATP, various concentrations of proteins and 10 nM (810 nM in nucleotides) of the Cy5-labeled oligonucleotide GO47. Experiments were performed as described below.

Binding of SSB proteins to ssDNA without (Figure 4A) or with (Figure 7B) Sak4 was assessed by mixing 2 μL of buffer G1 (20 mM Tris-HCl pH 8, 160 mM NaCl, 20% glycerol) containing or not various amounts of SSB with 16 μL of buffer G3 (12.5 nM of GO47, 12.5 mM Tris-HCl pH 7.5, 6.25 mM MgCl_2_, 1.25 mM ATP, 187.5 mM NaCl, 0.12 mg/mL BSA, 1.25 mM DTT) and incubated for 15 min at 30°C. Two μL of buffer G2 (10 mM Tris-HCl pH 8, 30 mM NaCl, 0.2 mM DTT, 20% glycerol), containing or not various amounts of Sak4, were added and solutions were incubated for an additional 15 min at 30°C. Sucrose was then added to a 6.5% final concentration prior to gel loading. Electrophoresis was performed with 5% acrylamide/bis-acrylamide (19:1) gel, 1X TAE migration buffer, at 120 V for 1 h, and bands were revealed by the BioRad ChemiDoc^TM^ MP imaging system.

The ATP-dependent ssDNA binding of Sak4 (Figure 1B) was assessed by mixing various amount of Sak4 (2 μL, in buffer G2) with 2 μL of buffer G1, 14 μL of 1.14X buffer G3 without ATP and 2 μL of water containing or not ATP (1 mM final concentration). Binding of Sak4 to dsDNA (Figure 1C) was identically assessed except that GO47 in buffer G3 was replaced by a dsDNA formed by the annealing of GO47 and GO34, prior to the experiment. Solutions were incubated for 15 min at 30°C and reactions were analyzed as described above.

### Single strand annealing assay

Unless otherwise stated, SSA assay was performed in a final volume of 20 μL of the buffer SSA (see above in section Gel shift assay) containing or not 1 mM ATP, various concentrations of proteins, 10 nM of GO47 and 10 nM of the complementary oligonucleotide GO34.

Effect of SSB proteins on the SSA activity of RecA and Sak4 (Figures 4B, 5) was assessed by mixing 2 μL of buffer G1 containing or not various amounts of SSB with 14 μL of buffer H1 (14.3 nM of the Cy5-labeled oligonucleotide GO47, 12.9 mM Tris-HCl pH 7.5, 6.4 mM MgCl_2_, 1.29 mM ATP, 193 mM NaCl, 0.13 mg/mL BSA, 1.29 mM DTT) and incubated 15 min at 30°C. Two μL of buffer G2, containing or not 18.5 μM RecA or 5 μM Sak4, were added and solutions were incubated for an additional 15 min at 30°C. The annealing reaction was started by addition of 2 μL of 100 nM GO34 in 0.78X buffer H1. After 30 min at 30°C, reactions were stopped by addition of 12 μL stop buffer 1 (10 mM Tris-HCl pH 7.5, 3 μM GO35 (untagged GO47), 0.5% SDS and 0.2 mg/mL proteinase K) and incubation for 15 min at 50°C. Sucrose was then added to a 10% final concentration prior to gel loading of half of the reaction. Electrophoresis was performed with a 10% acrylamide/bis-acrylamide (19:1) gel in 1X TBE buffer, at 120 V for 1 h, and the gel was revealed by the BioRad ChemiDoc^TM^ MP imaging system.

Requirement of ATP on the SSA activity of Sak4 in the presence of SSB_HK620_ (Figure 6) was identically done except that ATP was omitted or not in buffer H1.

SSA activity of Sak4 or GST alone (Figures 2A,B,D and Supplementary Figure S2) was assessed by incubating various amount of Sak4 or GST (2 μL, in buffer G2) with 2 μL of buffer G1 and 14 μL of buffer H1 with or without ATP (1 mM final concentration, as indicated) for 15 min at 30°C. The annealing reaction was started by addition of 2 μL of 0.78X buffer H1 containing or not 100 nM GO34 and with or without ATP. Solutions were incubated for 30 min at 30°C and reactions were analyzed as described above. *In vitro* time course formations of dsDNA by Sak4 (Figure 2C) were identically performed except that the final reaction volume was 180 μL (usual volumes multiplied by 9). Aliquots of 20 μL were taken at different times of incubation and reactions were stopped and analyzed as described above.

Although Sak4 required 150–200 mM NaCl for its SSA activity, RecA, Redβ and UvsX activities were progressively diminished with increasing NaCl concentration (not shown). These recombinases were therefore tested as follows (Figure 2D): GO47 (10 nM) was preincubated with variable amounts of protein for 2 min at 30°C (20 μL final volume) in buffer H2 (10 mM Tris-HCl pH 7.5, 5 mM MgCl_2_, 1 mM DTT, 0.1 mg/mL BSA). All reactions with UvsX contained 1 mM ATP. NaCl was provided by protein addition (10–15 mM). GO34 (10 nM) was then added. After 7 min of incubation at 30°C, reactions were stopped and DNA was analyzed as described above.

### Gel filtration assay

Sak4 (3.5 nmol), SSB_HK620_ (6.7 nmol) or SSB_HK620_Δ6 (6.7 nmol) were incubated alone or together in 1 mL of buffer B1 supplemented with 150 mM NaCl for 20 min at 30°C. After 5 min of centrifugation at 13,000 g, the soluble fraction was injected on a Superdex 200 10/300 GL (GE Healthcare) equilibrated in the same buffer. Twenty two fractions of 500 μL were collected, ranging from approximately 700 kDa (the void volume) to 10 kDa. Twenty μL of these fractions were analyzed by 12.5% SDS-PAGE and Coomassie blue staining.

### Cross-linking assay

Purified Sak4, SSB_HK620_ and SSB_HK620_Δ6 were dialyzed against 20 mM Hepes pH 7.5, 150 mM NaCl and 50% glycerol. Two microgram of each protein were incubated alone or together (as indicated in Supplementary Figure S7) in the same buffer supplemented or not with ATP 1 mM. After 30 min at 20°C, proteins were cross-linked or not for 20 min at 20°C with 1 mM of a fresh solution of Sulfo-EGS (Piercenet). Reactions were quenched by addition of 100 mM Tris-HCl pH 7.5 and proteins were denatured in a SDS reducing buffer (62.5 mM Tris-HCl pH 6.8, 2% SDS, 10% glycerol, 0.001% bromophenol blue and 100 mM DTT) at 95°C for 5 min. Multimerization of proteins was estimated by SDS-PAGE using a 12.5% polyacrylamide gel followed by Coomassie blue staining.

### Strand exchange assay

The strand exchange activity of RecA and Sak4 were assessed in a final reaction volume of 20 μL of buffer SE (14 mM Tris-HCl pH 7.5, 5 mM MgCl_2_, 22 mM NaCl, 1 mM DTT, 0.1 mg/mL BSA, 1 mM ATP, 10 mM phosphocreatine and 10 units/mL of creatine kinase). This assay was performed in successive steps as follows: 2 μL of buffer G2 containing or not 18.5 μM of RecA were mixed with 2 μL of buffer G2 containing or not 18.5 μM of Sak4, 10 μL of buffer H3 (20 mM Tris-HCl pH 7.5, 10 mM MgCl_2_, 2 mM DTT, 0.2 mg/mL BSA, 2 mM ATP, 40 mM phosphocreatine and 20 units/mL of creatine kinase) and 2 μL of circular ssDNA from ΦX174 (final concentration of 10 μM in nucleotides). Reaction mixtures were incubated for 10 min at 37°C. Two μL of buffer G1 containing or not 5 μM of *E. coli* SSB or SSB_HK620_ were added to the solution, and a second pre-incubation step was applied, at 37°C for 10 min. Strand exchange reaction was started by adding 2 μL *PstI*-linearized dsDNA form of ΦX174 (final concentration of 10 μM in nucleotides). After 30 min of incubation at 37°C, reactions were stopped by adding 6.7 μL of stop buffer 2 (5 mg*/*mL proteinase K, 2% SDS and 0.1 M EDTA) and incubating at 37°C for 30 min. 8.9 μL of DNA loading buffer (33% glycerol and 0.25% xylene cyanol) was added, reactions were cooled on ice, centrifuged 1 min at 14,000 g and half of the reaction volume was loaded for each sample on a 0.8% agarose gel in 1X TAE buffer. Electrophoresis was run at 4°C for 15 h at 25 V. The gel was incubated in 1X TAE buffer containing SYBR-Gold reagent (Thermofisher) and DNA bands were revealed by the BioRad ChemiDoc^TM^ MP imaging system.

## Author contributions

GH, AB, OS, SM, and FL performed the experiments. GH, RG, M-AP, FO, and FL designed and interpreted the experiments. M-AP and FL wrote the manuscript.

### Conflict of interest statement

The authors declare that the research was conducted in the absence of any commercial or financial relationships that could be construed as a potential conflict of interest.
