# Supplementary material for: Sak4 of Phage HK620 Is a RecA Remote Homolog With Single-Strand Annealing Activity Stimulated by Its Cognate SSB Protein
Source: Front Microbiol. 2018 Apr 24;9:743. doi: 10.3389/fmicb.2018.00743 (PMC5928155; doi:10.3389/fmicb.2018.00743)
Supplement: Supplementary file 1 [file Table_1.DOCX]

| strain | genetic background | plasmid | recombinational genes on plasmid | Origin |
| --- | --- | --- | --- | --- |
| MAC1774 | AB1157 *mutS*:specR *catss* kanR | none |  | This study |
| MAC1802 | MAC1774Δ*recA*306 *srl*:*Tn*10 tetR | none |  | This study |
| MFD*pir* | MG1655 RP4-2-Tc::[ΔMu1::aac(3)IV-ΔaphA-Δnic35-ΔMu2::zeo] ΔdapA::(erm-pir) ΔrecA | none |  | Ferrières et al., 2010 |
| MAC1628 | MFD*pir* | pJA3 |  | This study |
| MAC1798 | MAC1802 | pJA17 | *hkaM* *sak4* *ssb*_HK620_ *abc2* | This study |
| MAC1801 | MAC1802 | pKD46 | *orf60a* *red*α *red*β *gam* | Datsenko & Wanner, 2000 |
| MAC1879 | MAC1802 | pJA4 | none | Lopes et al., 2010 |
| MAC2089 | MAC1802 | pGH3 | *sak4* | This study |
| MAC1894 | MAC1802 | pJA12 | redβ | This study |
| MAC1895 | MAC1802 | pGH19 | *sak4* *ssb*_HK620_ | This study |
| MAC1897 | MAC1802 | pGH20 | *sak4* *abc2* | This study |
| MAC1922 | MAC1802 | pGH21 | *ssb*_HK620_ | This study |
| MAC2134 | MAC1802 | pOS10 | *sak4* *ssb*_HK620_Δ6 | This study |
| MAC2090 | MAC1774 | pGH3 | *sak4* | This study |
| MAC2091 | MAC1774 | pGH19 | *sak4* *ssb*_HK620_ | This study |
| ER2566 | F^-^ λ^-^ *fhu*A2 *ompT* *lacZ*::T7pol *gal* *sulA*11 Δ(*mcrC-mrr*)114 *mcr*-73 *zgb*-210 *endA*1 | none |  | NEB |
| MAC1987 | ER2566 Δ*recA*306 *srl*:*Tn*10 | none |  | This study |
| MAC1991 | MAC1987 | pSMG274 | *sak4* | This study |
| MAC2001 | MAC1987 | pSMG288 | *sak4* *ssb*_HK620_ | This study |
| HME57 | W3110 Δ(argF-lac)U169 *galK*_tyr145UAG_ | none |  | Datta et al., 2006 |
| G205 | HME57 Δ*recA306 srl*::*Tn*10 | none |  | This study |
| G209 | G205 | pKD46 | *orf60*a *red*α *red*β *gam* | This study |
| MAC1870 | G205 | pGH3 | *sak4* | This study |
| MAC1872 | G205 | pGH19 | *sak4* *ssb*_HK620_ | This study |
| MAC1494 | MG1655 Δ*recA*306 *srl*:*Tn*10 |  |  | This study |
| MAC2080 | MAC1494 | pJA192 | *recA* | This study |
| MAC2081 | MAC1494 | pGH19 | *sak4* *ssb*_HK620_ | This study |
| MAC2140 | MAC1494 | pGH21 | *ssb*_HK620_ | This study |
| MAC2142 | MAC1494 | pGH3 | *sak4* | This study |

**Supplementary Table S1.** Strains list.
