# Supplementary material for: Sak4 of Phage HK620 Is a RecA Remote Homolog With Single-Strand Annealing Activity Stimulated by Its Cognate SSB Protein
Source: Front Microbiol. 2018 Apr 24;9:743. doi: 10.3389/fmicb.2018.00743 (PMC5928155; doi:10.3389/fmicb.2018.00743)
Supplement: Supplementary file 2 [file Table_2.DOCX]

| Name/ gene targeted | Sequence |
| --- | --- |
| Maj98^1^  *catss* | CAACTTCTTCGCCCCCGTTTTCACCATGGGCAAATATTATACG**C**AAGGCGACAAGGTGCTGATGCCGCTGGCGATTCAGGT |
| 144^2^ *galK* | AAGTCGCGGTCGGAACCGTATTGCAGCAGCTTTA**C**CATCTGCCGCTGGACGGCGCACAAATCGCGCTTAA |
| Maj106^3^ *rpoB* | GTCAGACCGCCTGGGCCGAGTGCGGAGATACGACGTTTGTCCGTAATCTCAGACAGCGGGTTGTTCTGGTCCATAAACTGA |
| GO34^4^  *catss* | CAACTTCTTCGCCCCCGTTTTCACCATGGGCAAATAGTATACGTAAGGCGACAAGGTGCTGATGCCGCTGGCGATTCAGGT |
| GO47^4^ *catss* | ACCTGAATCGCCAGCGGCATCAGCACCTTGTCGCCTTACGTATACTATTTGCCCATGGTGAAAACGGGGGCGAAGAAGTTG |
| Maj99^1^  *catss* | CAACTTCTTCGC**G**CCCGTTTTCAC**T**ATGGGCAAATA**T**TATACG**C**AAGGCGACAAGGTGCTGATGCC**C**CTGGCGATTCAGGT |
| Maj100^1^  *catss* | CAACTT**T**TTCGC**G**CCCGT**C**TTCAC**T**ATGGGCAAATA**T**TATACG**C**AAGGCGA**T**AAGGTGCT**C**ATGCC**C**CTGGC**C**ATTCAGGT |
| Maj103^1^  *catss* | CAACTT**T**TT**T**GC**G**CCCGT**C**TTCAC**T**ATGGG**G**AAATA**T**TATACG**C**AAGG**G**GA**T**AAGGTGCT**C**ATGCC**C**CTGGC**C**ATTCAGGT |
| Maj101^1^  *catss* | CAACTT**T**TT**T**GC**G**CCCGT**C**TT**T**AC**T**ATGGG**G**AAATA**T**TATACG**C**AAGG**G**GA**T**AAGGT**C**CT**C**ATGCC**C**CT**C**GC**C**ATTCAGGT |
| Maj102^1^  *catss* | CAA**T**TT**T**TT**T**GC**G**CC**G**GT**C**TT**T**AC**T**ATGGG**G**AA**G**TA**T**TA**C**ACG**C**AAGG**G**GA**T**AA**A**GT**C**CT**C**ATGCC**C**CT**C**GC**C**AT**C**CA**A**GT |

^1^

**Supplementary Table S2.** Oligonucleotides for *in vivo* recombineering and *in vitro* SSA assays. ^1^ Red letters highlight the mismatches relative to the *catss* gene. ^2^ Described in Datta et al. 2006. Blue letter highlights a mismatch relative to the *galK* gene. ^3^ Green letter highlights a mismatch relative to the *rpoB* gene.^4^ For *in vitro* assays, GO47 is a Cy5 5’-labelled oligonucleotide complementary to GO34.
