# Supplementary material for: Sak4 of Phage HK620 Is a RecA Remote Homolog With Single-Strand Annealing Activity Stimulated by Its Cognate SSB Protein
Source: Front Microbiol. 2018 Apr 24;9:743. doi: 10.3389/fmicb.2018.00743 (PMC5928155; doi:10.3389/fmicb.2018.00743)
Supplement: Supplementary file 3 [file Table_3.DOCX]

| name | sequence |
| --- | --- |
| GO18 | ACCACTCCATGGGAATGAGTACTGCACTCGCAAC |
| GO20 | ACCACTCCATGGGAATGTCTGATTTAAAATCTCGTTTGATTAAAGCTTCTAC |
| GO22 | ACCACTCCATGGGAATGGGAACTGCGACATTAAT |
| GO24 | ACCACTCCATGGGAATGCAAATGTCGCAATTAAA |
| GO26 | CAAATTGGATCCTATGCTGCCACCTTCTGCTCTG |
| GO27 | CAAATTGGATCCTATTCATTAAATTCTTCCATATCACTTAGCTGTTCGAGGTC |
| GO28 | CAAATTGGATCCTATGCGGCGTTTTCCTTAATTT |
| GO29 | CAAATTGGATCCTATTGCATCGCTTTCGCGAACA |
| GO48 | ACCACTCCATGGGAATGAGCAACGTGATTTTTAC |
| GO49 | CAAATTGGATCCTAGAAAGGAGGGTAATCGTTCT |
| GO50 | ACCACTCCATGGGAATGCAACTTATCCAAGCGTT |
| GO51 | CAAATTGGATCCTAACGCTGGCCCCAAGGTGCCG |
| J55 | GGAATTCCGGAGGTATAAAACGAATGAGT |
| J56 | CATGCCATGGCATGTCATGCTGCCACCTTCTGCT |
| J63 | CCCCCGAGCTCGCAGGAGTAAAAATGGCTATC |
| J64 | GGGCCCCCTCAGCTTAAAAATCTTCGTTAGTTTC |
| J67 | CATGCCATGGCATGTCAGTCATTACTGATAGCGC |
| J68 | CCGGAATTCGGAGGCAAAATGATTCCGGTAGAACTGGC |
| J80 | CCGGAATTCGGAGGATGGGAACTGCGACATTAAT |
| J81 | ATGCCATGGTCATGCGGCGTTTTCCTTAA |
| J84 | GACGTGCACGTAAGAGGTTCCAACTTT |
| J85 | GCACCTTGTCGCCTTACGTATACTATTTGCCCATGGT |
| J86 | ACCATGGGCAAATAGTATACGTAAGGCGACAAGGTGC |
| J87 | CACGTCCTTTCGAATTTCTGCCATTCAT |
| Maj41 | CAGTGAAGCATCAAGACTAAC |
| Maj80 | ATGTATCAGCCTGATTTTCCTCCTGTACCTTTTCGTTCAGGACTGTACGCTTGCATGCAGATTGCAGC |
| Maj81 | TTAAACAGGATCTGCATTGCTTCCTCCGCATACCGGGCAACCACTGGCCCATATGAATATCCTCCTTA |
| Maj110 | CCATTACCATGGTCAGAAAGGAGGGTAATCGTT |
| Maj111 | CCATTACCATGGATTGTCATTACTGATAGCGCCATA |
| Maj112 | ATGGCACCATGGTACTGAAAATAAGGCTCCCATT |
| Maj113 | CCGGAATTCGGAGGCAAATTATGGGAACTG |
| Maj116 | CCGGAATTCGGAGGACGCCGCATGAGCAACGTG |
| Maj117 | GTCCATGG TTAACGCTGGCCCCAAGGTG |
| Maj118 | GTCCATGGTAGGAGGCAGATGCAACTTATCCAAGCGTTCAAC |
| Maj141 | GGGGAATTGTGAGCGGATAAC |
| Maj147 | TTACTCGAGTCACTGGCTGTAACCATAATCATC |
| OSMG438 | CAAAAAACCCCTCAAGACCC |
| OSMG568 | AAACATATGGGAACTGCGACATTAAT |
| OSMG571 | AAACATATGAGCAACGTGATTTTTAC |
| OSMG572 | TTTCTCGAGTCAGAAAGGAGGGTAATCGTT |
| OSMG573 | AAACATATGCAACTTATCCAAGCGTTCA |
| OSMG574 | TTTCTCGAGTTAACGCTGGCCCCAAGGTGCCG |

**Supplementary Table S3.** Oligonucleotides for plasmid and strain constructions.
