# Supplementary material for: Sak4 of Phage HK620 Is a RecA Remote Homolog With Single-Strand Annealing Activity Stimulated by Its Cognate SSB Protein
Source: Front Microbiol. 2018 Apr 24;9:743. doi: 10.3389/fmicb.2018.00743 (PMC5928155; doi:10.3389/fmicb.2018.00743)
Supplement: Supplementary file 4 [file Table_4.DOCX]

| plasmid name | plasmid description | insert | | | | vector | | origin |
| --- | --- | --- | --- | --- | --- | --- | --- | --- |
|  |  | gene amplified by PCR or obtained after digestion | DNA matrix | PCR ol. 1 or enzyme | PCR ol. 2 or enzyme | original plasmid | digested by |  |
| pKD46 | Para:*module*_λ_ |  |  |  |  | pSC101 origin, AmpR |  | Datsenko & Wanner, 2000 |
| pJA4 | No insert |  |  |  |  | pKD46 | Δ(EcoRI NcoI) | Lopes et al., 2010 |
| pGH3 | Para: *sak4* | *sak4*_HK620_ | HK620 | J80 | Maj113 | pKD46 | EcoRI and NcoI | this study |
| pGH19 | Para:*sak4*+*ssb*_HK620_ | *sak4*+*ssb*_HK620_ | pJA17 | Maj110 | Maj113 | pKD46 | NcoI &BamHI | this study |
| pOS10 | Para:*sak4*+*ssb*_HK620_Δ6 | *ssb*_HK620_Δ6 | pOS8 |  |  | pGH19 | NdeI and StyI | this study |
| pGH20 | Para:*sak4*+*abc2*_HK620_ | *abc2*_HK620_ | pJA17 | Maj111 | Maj112 | pGH3 | NcoI | this study |
| pGH21 | Para:*ssb*_HK620_ | *ssb*_HK620_ | HK620 | Maj41 | Maj116 | pKD46 | EcoRI and NcoI | this study |
| pJA16 | Para:*redβ* | *redβ* | pKD46 | J55 | J56 | pKD46 | EcoRI and NcoI | this study |
| pJA17 | Para:*module*_HK620_ | *module*_HK620_ | HK620 | J67 | J68 | pKD46 | EcoRI and NcoI | this study |
| pJ192 | Para:*recA* | *recA* | MG1655 | J63 | J64 | pKD46 | SacI BbvCI | this study |
| pJA3 | oriV R6K γ oriT RP4 lacZbeg-CmR-lacZend |  |  |  |  |  |  | Amarir-Bouhram et al., 2011 |
| pJ411 | pBR322 origin, KanR, pT7 |  |  |  |  |  |  | Menlo Park, CA |
| pSMG274 | PT7: *sak4* | *sak4* | pGH6 | OSMG568 | OSMG438 | pJ411 | NdeI and XhoI | this study |
| pSMG279 | PT7: *ssb*_HK620_ | *ssb*_HK620_ | pGH21 | OSMG571 | OSMG572 | pJ411 | NdeI and XhoI | this study |
| pSMG288 | PT7: *sak4*_+_*ssb*_HK620_ | *sak4*+*ssb*_HK620_ | pSMG274 | AscI | BamHI-Klenow | pSMG279 | AscI and BsaI-Klenow | this study |
| pOS8 | PT7: *ssb*_HK620_Δ6 | *ssb*_HK620_Δ6 | pSMG279 | Maj141 | Maj147 | pSMG279 | BsaI and XhoI | this study |
| pETM30 | pBR322 origin, KanR, pT7:GST |  |  |  |  |  |  | NE Biolabs |
| pGH5 | PT7: GST-*uvsX* | *uvsX* | T4 | GO20 | GO27 | pETM30 | NcoI &BamHI | this study |
| pGH11 | PT7:GST-*redβ* | *redβ* | pKD46 | GO18 | GO26 | pETM30 | NcoI &BamHI | this study |

**Supplementary Table S4.** Plasmid constructions.
