# Supplementary material for: Sak4 of Phage HK620 Is a RecA Remote Homolog With Single-Strand Annealing Activity Stimulated by Its Cognate SSB Protein
Source: Front Microbiol. 2018 Apr 24;9:743. doi: 10.3389/fmicb.2018.00743 (PMC5928155; doi:10.3389/fmicb.2018.00743)
Supplement: Supplementary file 5 [file Table_5.DOCX]

| **Accession number** | [Phage name](javascript:__doPostBack('resultat','Sort$PHAGENAME')) | [Host](javascript:__doPostBack('resultat','Sort$HOST')) | Phage lifestyle* | Sak4, gene name | Length (AA) | SSB  gene name | Exonuclease or Abc2 | Other recombination function in the vicinity |
| --- | --- | --- | --- | --- | --- | --- | --- | --- |
| [NP_510911](http://www.ncbi.nlm.nih.gov/protein/NP_510911) | phi ETA | *Staphylococcus aureus* | T | ORF17 | 259 | ORF18 | ? | NinB- domain |
| [YP_240428](http://www.ncbi.nlm.nih.gov/protein/YP_240428) | 71 | *Staphylococcus aureus* | T | Orf16 | 262 | ORF25 | ? | NinB- domain |
| [YP_240651](http://www.ncbi.nlm.nih.gov/protein/YP_240651) | 52A | *Staphylococcus aureus* | T | Orf16 | 259 | ORF22 | ? | NinB- domain |
| [YP_240583](http://www.ncbi.nlm.nih.gov/protein/YP_240583) | 29 | *Staphylococcus aureus* | T | Orf17 | 262 | ORF24 | ? | NinB- domain |
| [YP_239540](http://www.ncbi.nlm.nih.gov/protein/YP_239540) | 187 | *Staphylococcus aureus* | T | gp53 | 257 | gp54 | ? | NinB- domain |
| [YP_239613](http://www.ncbi.nlm.nih.gov/protein/YP_239613) | 69 | *Staphylococcus aureus* | T | Orf15 | 262 | Orf24 | ? | NinB- domain |
| [NP_958639](http://www.ncbi.nlm.nih.gov/protein/NP_958639) | 77 | *Staphylococcus aureus* | T | Orf38 | 262 | Orf39 | ? | NinB- domain |
| [YP_240507](http://www.ncbi.nlm.nih.gov/protein/YP_240507) | 55 | *Staphylococcus aureus* | T | Orf17 | 257 | Orf24 | ? | NinB- domain |
| [YP_240351](http://www.ncbi.nlm.nih.gov/protein/YP_240351) | ROSA | *Staphylococcus aureus* | T | Orf14 | 262 | Orf22 | ? | NinB- domain |
| [YP_240275](http://www.ncbi.nlm.nih.gov/protein/YP_240275) | 96 | *Staphylococcus aureus* | T | Orf14 | 259 | Orf21 | ? | NinB- domain |
| [YP_873963](http://www.ncbi.nlm.nih.gov/protein/YP_873963) | phiNM | *Staphylococcus aureus* | U | gp14 | 262 | Gp15 | ? | NinB- domain |
| [YP_001429977](http://www.ncbi.nlm.nih.gov/protein/YP_001429977) | tp310-3 | *Staphylococcus aureus* | U | gp15 | 259 | gp16 | ? | NinB- domain |
| [YP_918908](http://www.ncbi.nlm.nih.gov/protein/YP_918908) | phiPVL | *Staphylococcus aureus* | U | gp18 | 257 | Gp19 | ? | NinB- domain |
| [YP_001004276](http://www.ncbi.nlm.nih.gov/protein/YP_001004276) | phiETA2 | *Staphylococcus aureus* | U | gp16 | 259 | Gp17 | ? | NinB- domain |
| [NP_803265](http://www.ncbi.nlm.nih.gov/protein/NP_803265) | phi 11 | *Staphylococcus aureus* | T | ORF12 | 259 | ORF13 | ? | NinB- domain |
| [NP_803367](http://www.ncbi.nlm.nih.gov/protein/NP_803367) | phi 13 | *Staphylococcus aureus* | T | ORF12 | 259 | ORF13 | ? | NinB- domain |
| [NP_049951](http://www.ncbi.nlm.nih.gov/protein/NP_049951) | Sfi19 | *Streptococcus thermophilus* | ex-T | p31 | 233 | P33 | ? | helicase and Mu-gam |
| [YP_238517](http://www.ncbi.nlm.nih.gov/protein/YP_238517) | 2972 | *Streptococcus thermophilus* | ex-T | gp35 | 233 | Gp36 | ? | helicase |
| [NP_049420](http://www.ncbi.nlm.nih.gov/protein/NP_049420) | DT1 | *Streptococcus thermophilus* | ex-T | p31 | 233 | P33 | ? | helicase |
| [NP_056708](http://www.ncbi.nlm.nih.gov/protein/NP_056708) | Sfi11 | *Streptococcus thermophilus* | ex-T | P38 | 233 | P40 | ? | helicase and Mu-gam |
| [NP_049998](http://www.ncbi.nlm.nih.gov/protein/NP_049998) | Sfi21 | *Streptococcus thermophilus* | T | p35 | 233 | P37 | ? | helicase and Mu-gam |
| [NP_695087](http://www.ncbi.nlm.nih.gov/protein/NP_695087) | O1205 | *Streptococcus thermophilus* | T | p09 | 233 | P11 | ? | helicase and Mu-gam |
| [NP_795445](http://www.ncbi.nlm.nih.gov/protein/NP_795445) | 315.2 | *Streptococcus pyogenes* | T | SpyM3_0962 | 226 | SpyM3_0960 | ? | helicase and Mu-gam |
| [NP_795615](http://www.ncbi.nlm.nih.gov/protein/NP_795615) | 315.5 | *Streptococcus pyogenes* | T | SpyM3_1344 | 226 | SpyM3_0964 | ? | helicase and Mu-gam |
| [NP_695186](http://www.ncbi.nlm.nih.gov/protein/NP_695186) | phig1e | *Lactobacillus* | T | p57 | 220 | P56 | ? | highly conserved NinB-HNH 2 domain |
| [NP_680513](http://www.ncbi.nlm.nih.gov/protein/NP_680513) | A2 | *Lactobacillus casei* | T | p34 | 240 | P37 | ? | helicase and Mu-gam |
| [YP_025055](http://www.ncbi.nlm.nih.gov/protein/YP_025055) | phi AT3 | *Lactobacillus casei* | T | ORF28 | 235 | ORF29 | ? | Mu-gam |
| [YP_223910](http://www.ncbi.nlm.nih.gov/protein/YP_223910) | phiJL-1 | *Lactobacillus plantarum* | ex-T | ORF224 | 224 | Not found | RecE |  |
| [YP_358787](http://www.ncbi.nlm.nih.gov/protein/YP_358787) | Lc-Nu | *Lactobacillus rhamnosus* | ex-T | ORF29 | 235 | ORF30 | RecE | Mu-gam |
| [NP_050126](http://www.ncbi.nlm.nih.gov/protein/NP_050126) | phi adh | *Lactobacillus gasseri* | T | P18 | 223 | P21 | ? | helicase |
| [NP_116538](http://www.ncbi.nlm.nih.gov/protein/NP_116538) | BK5-T | *Lactococcus lactis* | T | p46 | 234 | Not found | ? | Mu-gam |
| [YP_001469088](http://www.ncbi.nlm.nih.gov/protein/YP_001469088) | KSY1 | *Lactococcus lactis* | U | gp89 | 246 | Not found | RecE | helicase |
| [NP_511030](http://www.ncbi.nlm.nih.gov/protein/NP_511030) | 2389 | *Listeria monocytogenes* | T | gp49 | 230 | Gp51 | ? | helicase and Mu-gam |
| [YP_001468696](http://www.ncbi.nlm.nih.gov/protein/YP_001468696) | B025 | *Listeria monocytogenes* | U | gp57 | 230 | Gp59 | ? | helicase and Mu-gam |
| [YP_655252](http://www.ncbi.nlm.nih.gov/protein/YP_655252) | PBI1 | *Mycobacterium smegmatis* | U | gp56 | 286 | Not found | RecE |  |
| [YP_655438](http://www.ncbi.nlm.nih.gov/protein/YP_655438) | PLot | *Mycobacterium smegmatis* | U | gp59 | 286 | Not found | RecE |  |
| [YP_224274](http://www.ncbi.nlm.nih.gov/protein/YP_224274) | phiHSIC | *Listonella pelagia* | U | ORF46 | 252 | ORF45 | RecE | helicase |
| [YP_001210265](http://www.ncbi.nlm.nih.gov/protein/YP_001210265) | BcepGomr | *Burkholderia cepacia* | U | gp45 | 220 | Gp44 | RecE | helicase |
| [NP_112045](http://www.ncbi.nlm.nih.gov/protein/NP_112045) | HK620 | *Escherichia coli* | T | HkaL | 226 | HkaK | abc2 |  |
| [YP_001293439](http://www.ncbi.nlm.nih.gov/protein/YP_001293439) | PA73 | *Pseudomonas aeruginosa* | U | ORF032 | 238 | ORF031 | RecE | helicase |
| [NP_852729](http://www.ncbi.nlm.nih.gov/protein/NP_852729) | Aaphi23 | *Actinobacillus*  *Actinomycetem-comitans* | T | p07 | 297 | Not found | RecE |  |
| [NP_818613](http://www.ncbi.nlm.nih.gov/protein/NP_818613) | Barnyard | *Mycobacterium smegmatis* | U | gp75 | 340 | Not found | RecE |  |
| [NP_839901](http://www.ncbi.nlm.nih.gov/protein/NP_839901) | 4268 | *Lactococcus lactis* | V | p10 | 306 | Not found | RecE |  |
| [YP_164392](http://www.ncbi.nlm.nih.gov/protein/YP_164392) | BCJA1c | *Bacillus clarkii* | T | gp14 | 362 | Not found | ? |  |
| [NC_008720](http://www.ncbi.nlm.nih.gov/nuccore/NC_008720) | N4** | *Escherichia coli* | V | Gp44 | 251 | Gp45 | ? |  |

**Supplementary Table S5.** List of the initial 44 phages encoding Sak4 and described in Lopes et al. (2010), indicating whether an SSB protein is present at the vicinity. *T :temperate, ex-T :ex-temperate, V : virulent, U : unknown. ** Genome not mentioned in Lopes et al. (2010). For an updated list, see Phagonaute web site, and PGC2 database (Delattre et al., 2016).
