# Supplementary material for: Sak4 of Phage HK620 Is a RecA Remote Homolog With Single-Strand Annealing Activity Stimulated by Its Cognate SSB Protein
Source: Front Microbiol. 2018 Apr 24;9:743. doi: 10.3389/fmicb.2018.00743 (PMC5928155; doi:10.3389/fmicb.2018.00743)
Supplement: Supplementary file 6 [file Table_6.DOCX]

| Strain | Back  ground | Gene induced | CmR (Maj98) | Gal+(144) | RifR (Maj106) |
| --- | --- | --- | --- | --- | --- |
| MAC1879 | MAC1802 | none | 2.2 (+/-2.3) 10^-8^ |  | 1.3 (+/- 0.1) 10^-5^ |
| MAC2089 | MAC1802 | *sak4* | 3.3 (+/-1.1) 10^-7^ |  |  |
| MAC1798 | MAC1802 | Module_HK620_ | 4.7 (+/-3.2) 10^-6^ |  |  |
| MAC1897 | MAC1802 | *sak4 abc2*_HK620_ | 2.8 (+/-2.5) 10^-7^ |  |  |
| MAC1895 | MAC1802 | *sak4 ssb*_HK620_ | 1.1 (+/-1.2) 10^-5^ |  |  |
| MAC1922 | MAC1802 | *ssb*_HK620_ | 3.0 (+/-2.4) 10^-8^ |  |  |
| MAC2134 | MAC1802 | *sak4 ssb*_HK620_Δ6 | 1.1 (+/-0.4) 10^-6^ |  |  |
| MAC1894 | MAC1802 | *red*β | 1.1 (+/-0.15) 10^-6^ |  |  |
| MAC1801 | MAC1802 | Module_λ_ | 5.4 (+/-4.7) 10^-6^ |  | 9.1 (+/- 6) 10^-4^ |
| MAC1774 | as MAC1802 but RecA+ | *recA** | 2.0 (+/-0.5) 10^-8^ |  |  |
| MAC2090 | MAC1774 | *sak4* | 2.3 (+/-1.5) 10^-7^ |  |  |
| MAC2091 | MAC1774 | *sak4 ssb*_HK620_ | 7.2 (+/-5.8) 10^-6^ |  |  |
| G205 | HME57 Δ*recA306 srl*::*Tn*10 | none |  | 5.5 (+/- 5.1) 10^-7^ | 2 10^-6^ |
| G209 | G205 | Module_λ_ |  | 2.2 (+/- 1.1) 10^-3^ | 1 10^-3^ |
| MAC1870 | G205 | *sak4* |  | 1.7 (+/- 1.2) 10^-6^ |  |
| MAC1872 | G205 | *sak4 ssb*_HK620_ |  | 2.0 (+/- 1.5) 10^-5^ |  |
| MAC1987 | As ER2566 but Δ*recA* |  |  |  | 8.1 (+/-8.2) 10^-6^ |
| MAC2001 | MAC1987 | *sak4* |  |  | 3.0 (+/-1.2) 10^-5^ |
| MAC1991 | MAC1987 | *sak4 ssb*_HK620_ |  |  | 1.7 (+/-1.0) 10^-4^ |

**Supplementary Table S6.** Recombineering efficiency at the *catss* locus (CmR), and comparison with *galK* (Gal^+^) and *rpoB* (RifR) loci. *the *recA* gene is not induced by arabinose, it is the wild type chromosomal gene.
