# Supplementary material for: Sak4 of Phage HK620 Is a RecA Remote Homolog With Single-Strand Annealing Activity Stimulated by Its Cognate SSB Protein
Source: Front Microbiol. 2018 Apr 24;9:743. doi: 10.3389/fmicb.2018.00743 (PMC5928155; doi:10.3389/fmicb.2018.00743)
Supplement: Supplementary file 8 [file Presentation_2.PDF]

Supplementary Figure S1. Purified proteins used in this study. SDS PAGE analyses of the different proteins used in this study stained with Coomassie blue. Redβ, UvsX, Sak4, SSB_{HK620} and $SSB_{HK620}\Delta_6$ were purified as described in the Materials and Methods section. *E. coli* RecA and SSB proteins were from Epicentre (Tebu-bio). Black bars indicate the position of the protein markers after migration and their molecular weight (in kDa).

Supplementary Figure S2. The GST protein does not influence the spontaneous annealing of two complementary ssDNA molecules. GO47 (10 nM) was pre-incubated for 15 minutes with variable concentrations of GST, as indicated below the gel, in a buffer containing 1 mM ATP. Reactions were started by addition of GO34 (10 nM) and incubated for 30 minutes at 30°C. Products of the reaction were analyzed on native acrylamide gels **(A)** and annealing was quantified as a function of the GST concentration **(B)**. ss and ds stand for single- and double-stranded DNA respectively.

Supplementary Figure S3. *ssb*$_{HK620}$ genome context. HkaK of phage HK620 was used as a query on Phagonaute (database pgc1) with a 98% confidence probability cutoff setting. Next to the *ssb* gene (red), the *sak4* gene (blue) is always found. Under the figure are the annotation taken from PFAM and ACLAME.

Supplementary Figure S4. Sak4 tolerance to mismatches. **(A)** Sak4 and Redβ tolerate up to 12% divergence *in vivo*. Recombineering was performed at the *cat*ss locus with increasingly diverged oligonucleotides, using 1 µg of oligonucleotides Maj99 (6 % divergence with *catss*), Maj100 (12 %) or Maj103 (16 %) to transform strain MAC1798 (“RecA-, moduleHK620”) or strain MAC1801 (“RecA-, module λ”). **(B)** For Sak4 and Redβ-mediated events, sequencing of 8 recombinants obtained at 12 % divergence. On top of the panel is the sequence of Maj100 with the central T and C in bold showing the two mismatches permitting correction of stop codons in *catss*. The red bases correspond to silent mutations introduced into Maj100 to create a 12% diverged sequence, relative to *cat*ss. “+” indicates mutations initially present in Maj100 that have been incorporated into the CmR recombinants, ”-“ unincorporated mutations. The grey regions cover the mismatches that have been integrated into the chromosome. **(C)** Sak4-mediated SSA tolerates up to 20 % diverged sequences. Annealing was tested between GO47 and oligonucleotides with increasing numbers of mismatches: GO34 (0 %), Maj98 (2 %), Maj99 (6 %), Maj100 (12 %), Maj101 (20 %) and Maj102 (28 %). Upper panel: representative gel obtained after annealing mediated by 1.85 µM of Sak4 in buffer containing 1 mM ATP for 45 minutes at 30°C. Lower panel: the mean values from at least three experiments of the spontaneous annealing (grey line) and of the annealing mediated by Sak4 (black line) in these conditions are given. Error bars indicate the standard deviation. **(D)** RecA (0.5 µM) and Redβ (2 µM) also tolerate 20% divergence for annealing (same conditions as in Figure 2D). GST (0.5 µM) was used as a control in these conditions. **(E)** UvsX (0.5 µM) anneals up to 28% diverged oligonucleotides, in an ATP-dependent reaction (same conditions as in Figure 2D, except the presence or absence of 1 mM ATP in the reaction).

Supplementary Figure S5. Deletion of the six terminal amino acids of SSB_{HK620} does not change the stoichiometry of the phage SSB. 6.7 nmol SSB_{HK620} or $SSB_{HK620}\Delta 6$ were injected on a Superdex 200HR10/30 gel filtration column equilibrated in buffer B1 supplemented with 150 mM NaCl (see Material and Methods). The OD_{280nm} was measured at the outlet of the column and is presented for each protein. Molecular weight (in kDa) of standard proteins used to calibrate the column (arrow heads) is indicated.

Supplementary Figure S6. SSB_{HK620} and the truncated $SSB_{HK620}\Delta 6$ inhibit spontaneous annealing of complementary ssDNA. **(A)** gel shift assay of ssDNA using full length and C-terminally truncated phage SSB proteins, as indicated above each gel, was done as described in the legend of Figure 4A. **(B)** SSA assay using SSB_{HK620} and $SSB_{HK620}\Delta 6$. Experiments were performed as described in the legend of Figure 4B, except that RecA was omitted.

Supplementary Figure S7. SSB_{HK620} does not form a multiprotein complex with Sak4 even in the presence of a crosslinking agent. Two µg of SSB_{HK620}, $SSB_{HK620}\Delta 6$ or Sak4 were incubated alone or together (as indicated above the gel) in a reaction buffer supplemented or not with 1 mM ATP (+ ATP or – ATP, respectively). Proteins were incubated (+) or not (-) with 1 mM of the crosslinker Sulfo-EGS prior to quenching and analysis by SDS-PAGE 12.5%. MW: Molecular weight markers (in kDa) indicated on the left. The amount of monomeric Sak4 treated with Sulfo-EGS in the presence or not of the phage SSB is similar and no new bands are visible when proteins were mixed together compared to proteins treated alone.

Supplementary Figure S8. Post-conjugative, homologous recombination assay. Plasmid pJA3 is unable to replicate in the recipient strain post conjugation. Its two 1kb regions of homology with *lacZ* on the recipient chromosome are shown in blue. The *cat* gene, conferring resistance to chloramphenicol, is placed between the two *lacZ* regions. CmR ex-conjugants are produced either by single (SCO) or double crossing-overs (DCO). DCO lead to lac-clones, and were found approximately in half of the CmR colonies.

Supplementary Figure S9. Sak4 does not stimulate nor inhibit the RecA-promoted strand exchange reaction. Assays were conducted as in Figure 8C, except that incubation time was reduced to 12 min. **(A)** Adding Sak4 at various concentrations (from the left to the right: 0.12, 0.23, 0.46, 0.93, 1.85 and 3.7 µM) does not modify the reaction mediated by 1.85 µM of RecA, regardless of the SSB (0.5 µM) added. **(B)** Effect of the order of addition. Adding Sak4 prior RecA (1 and 2 in the columns corresponding to Sak4 and RecA, respectively), RecA prior to Sak4 (1 and 2 in the columns corresponding to RecA and Sak4, respectively) or both proteins together (last line) to the ssc substrate did not modify the reaction.
